# Uncertainty Quantification for *In Silico* Chemistry

**DOI:** 10.1021/acs.chemrev.5c00931

**Published:** 2026-03-04

**Authors:** Tom Frömbgen, Elizaveta Surzhikova, Jürgen Dölz, Jonny Proppe, Barbara Kirchner, Christoph R. Jacob

**Affiliations:** † Mulliken Center for Theoretical Chemistry, Clausius-Institute for Physical and Theoretical Chemistry, 9374 University of Bonn, Beringstraße 4, 53115 Bonn, Germany; ‡ Institute of Physical and Theoretical Chemistry, 26527 Technische Universität Braunschweig, Gaußstr. 17, 38106 Braunschweig, Germany; ¶ Institute for Numerical Simulation, University of Bonn, Friedrich-Hirzebruch-Allee 7, 53115 Bonn, Germany

## Abstract

The rapid growth of worldwide computing power has transformed *in silico* chemistry into a discipline that is integrated into the daily work of many chemists. Nowadays, researchers find it increasingly straightforward to predict a wide range of molecular properties and chemical processes at reasonable computational cost. The resulting abundance of data generated by quantum chemistry, molecular dynamics simulations, and chemical machine learning naturally raises questions about accuracy, precision, and reliability as well as the systematic treatment of errors and uncertainties. Addressing these questions through rigorous mathematical frameworks is at the heart of uncertainty quantification. In the past years, the incorporation of uncertainty quantification into *in silico* chemistry has gained attention, motivated by its ability to provide deeper insights into chemical phenomena. In this review, we establish a common language for uncertainty quantification with respect to *in silico* chemistry, introduce the key mathematical formalisms, and survey the growing body of work that applies uncertainty quantification across different areas of *in silico* chemistry.

## Graphical abstract

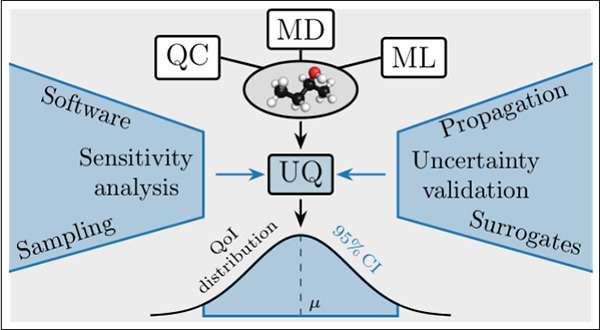

## 1 Introduction

A preprint of this work was previously published at https://chemrxiv.org/.^1^

In physics one is constantly faced with the task of having to draw conclusions from imperfect information.^2^

Computational chemistry and the associated software have reached a level of maturity where even novices or nonexperts in the field can perform calculations, conduct computer-based experiments, and generate data. At the same time, scientific targets are becoming increasingly complex, often demanding significant computational resources to uncover new insights into systems or phenomena that are not yet (fully) understood.

Examples posing such challenges include modeling large ensembles of molecules or interfaces and performing high-accuracy quantum-chemical calculations, which require substantial sampling and/or large basis sets. Furthermore, the desire to tackle more complex chemical systems is frequently hindered by a lack of sufficient information or input data, casting doubt on the reliability of highly accurate calculations. Therefore, the resulting data are often far from being perfect and carry uncertainties that modern *in silico* chemistry should take into account. In that sense, the expressive motto from the review article of von Toussaint^2^ on Bayesian methods in physics seamlessly translates to *in silico* chemistry.

The aforementioned aspects are illustrated by the transformation the daily routine of *in silico* chemistry has taken. For example, in ionic liquid research,^3–5^ a couple of decades ago, research focused on studying structures or component specification with sophisticated force fields in molecular dynamics simulations or intermolecular interactions in quantum-chemical calculations between one cation and one anion.^6–15^ Nowadays, more involved multicomponent mixtures or transport properties like diffusion coefficients or ionic conductivities are considered.^16–19^ This requires longer simulation times, larger system sizes, and also more refined force fields.^20–22^ To deal with future requirements and applied topics, e.g., deep eutectic solvents,^23–25^ gas absorption,^26^ or energy storage materials^17,19,27,28^ additional phenomena such as electric fields, solid–liquid interfaces, and growth phenomena need to be accounted for, adding a layer of complexity and dependence on possibly inexact information.

A similar transformation can be observed in the field of reaction mechanism exploration. In the past, computational studies were often limited to isolated reaction steps, treating only reactants, transition states, and products within a single elementary reaction. With increasing computational power and more sophisticated algorithms, researchers now construct complex reaction networks where each node represents not just a single structure but an ensemble of conformations. Automated approaches have been developed to construct complex reaction networks and explore reaction mechanisms for numerous reactant molecules.^29–31^ The complexity of these networks can be further increased by introducing environment effects such as microsolvation.^32,33^

Beyond reaction networks, similar advancements have transformed other domains of computational chemistry. The study of catalytic systems, for example, has shifted from static models of active sites to dynamic simulations incorporating explicit solvent, surface reconstructions, and even operando conditions.^34,35^ Machine learning methods now complement traditional quantum-chemical calculations by predicting activation energies,^36^ optimizing reaction pathways,^37^ or accelerating molecular dynamics simulations.^38–40^

A particularly pressing challenge for the future is the accurate and efficient description of excited-state processes, which are critical in fields ranging from photochemistry and spectroscopy to optoelectronic materials. While current methods provide valuable insights, they are often computationally prohibitive when applied to large or complex systems. The ability to construct and analyze potential energy surfaces that span multiple electronic states would mark a major milestone in theoretical chemistry, further pushing the boundaries of what computational approaches can achieve. Recent developments in computational methods have enabled the efficient calculation of excited states, allowing reaction networks to represent multiple potential energy surfaces.^41,42^

However, all such applications of *in silico* chemistry to ever more complex systems rely on imperfect models and the resulting data is prone to uncertainties. When dealing with imperfect data, it is key to acknowledge the fact that imperfect data require appropriate statistical treatment to quantify the level of “imperfectness”. By taking a step back from the illusion of perfection, and by applying techniques from uncertainty quantification (UQ), even more beneficial conclusions can be drawn. As a valuable starting point for uncertainty quantification, the following definition of Molinero and co-workers can be considered:

Uncertainty quantification (UQ) describes the process of quantifying uncertainties and how they are propagated in a model system of interest, such as a molecular simulation.^43^

Following this process and drawing appropriate conclusions should be one of the key components of any scientific workflow to assess the quality of the scientific results.

The assessment of uncertainties in experimental sciences is well-established. The most basic and general work is provided by Evaluation of Measurement Data—Guide to the Expression of Uncertainty in Measurement (GUM).^44^ It is an international standard for quantification of uncertainties independent of the methods applied. An easier-to-digest work is provided in the Guidelines for Evaluating and Expressing the Uncertainty of NIST Measurement Results (NIST Technical Note 1297).^45^ This work is comparable to GUM, but less general and mainly focused on experiments. For chemistry, there is a IUPAC guide for quantifying uncertainties in experimental measurements,^46^ and basic requirements for reporting experimental uncertainties are provided, e.g., in guidelines of scientific journals such as *The Journal of Physical Chemistry*.^47^

While many principles from the experimental sciences can also be applied in the computational sciences, the latter often require different strategies for UQ.^48^ The mathematical foundations of uncertainty quantification in computer simulations have been well investigated in the last decades, see for example the several mathematics-centered books^49–52^ and reviews^53–58^ that are available. These mathematical tools have become an indispensable part of different scientific disciplines, such as climate modeling and engineering.^59–62^

In the field of *in silico* chemistry, the assessment of uncertainties is less well-established, and has only been emerging rather recently. For overview articles highlighting the importance of uncertainty quantification in computational chemistry, we refer the reader to refs 63–70 . Here we review applications of UQ in different areas of *in silico* chemistry, covering quantum chemistry, molecular dynamics simulations, and chemical machine learning. We will address UQ in materials modeling^71^ only as far as it directly relates to atomistic simulations. Also, UQ for quantum computing with applications to *in silico* chemistry is beyond the scope of this review, and the reader is kindly referred to other works and reviews.^72–76^

The above quote by Molinero and co-workers highlights that uncertainty quantification is a multistep workflow. As a minimal or initial step, UQ involves identifying the stages at which uncertainties can enter a computation, such as through uncertain parameters. At a more advanced level, it requires assessing how these uncertainties propagate through the computation, ultimately affecting the quantity of interest (QoI). Additional insights into QoIs can be gained by comparing them to highly accurate reference values (referred to as benchmarks) to quantify the uncertainties.

We stress here that the latter step is optional, and while this requires benchmark values to compare with, the aim of UQ is different from those of benchmark calculations. UQ helps to understand how actionable conclusions can be drawn from current state-of-the-art chemical computations—an endeavor that may still lead to an added value, despite possible shortcomings of the calculations. This in turn leads to another step of UQ, where UQ can guide researchers to improved approaches, for example with inverse UQ or multifidelity methods. Thus, UQ clearly holds the potential for novel method developments.

Moreover, besides basic research interest, there is also the potential for the development of novel approaches that UQ permits, as will be shown in this review. UQ can also serve as a control mechanism to counteract faulty data production and misconduct behavior.^77,78^ In view of the ever-increasing amount of data produced as well as the growing capabilities of artificial intelligence (AI) and machine learning (ML)^79,80^ in almost all scientific research fields, UQ constitutes a cornerstone of control mechanisms to maintain scientific quality and reproducibility.^81,82^

Similarly, as outlined in this review, UQ is not at all restricted to machine learning albeit being an important and necessary part of it—and vice versa—which is why this review should not be subsumed under many excellent reviews about machine learning in chemistry,^41,83–91^ but contain some of the work that strongly focus on UQ aspects.

In this review, we establish a common language for uncertainty quantification with respect to *in silico* chemistry, introduce the key mathematical formalisms, and survey the growing body of work that applies uncertainty quantification across different areas of *in silico* chemistry.

We first establish a common language for uncertainty quantification with respect to *in silico* chemistry in section 2 by presenting key terms and definitions in section 2.1, discussing the most important sources of uncertainty in section 2.2, and demonstrating the UQ workflow in section 2.3. This is followed by a rigorous introduction of basic mathematical principles on a level that should be understandable to *in silico* chemists in section 3: we address the modeling of uncertainties in computations (section 3.1), problem classes of UQ (section 3.2), sensitivity analysis (section 3.3), sampling methods (section 3.4), surrogate models (section 3.5), uncertainty validation (section 3.6), dimension reduction (section 3.7) and provide a brief overview on UQ software with potential applications to *in silico* chemistry (section 3.8). Section 4 deals with the applications of UQ to *in silico* chemistry, focusing on uncertainties in energies in section 4.1, in trajectories and other simulation-based properties in section 4.2 and in static properties and electronic structure applications in section 4.3. We close this review with recommendations and conclusions in section 5.

## 2 Taxonomy of Uncertainty Quantification

### 2.1 Key Terms and Definitions

This section introduces core concepts and definitions for clear and easier understanding of meaningful uncertainty quantification for *in silicio* chemistry.

• **Quantity of interest** is the particular quantity that is going to be calculated and that is affected by the uncertainty. This is usually an output quantity of a calculation, rather than an input quantity.

• **Reference data** or **reference values** represent the best available estimate of the true values and are usually themselves uncertain. They may be derived from high-quality experimental data or from more accurate or established computational methods.

• **Error** refers to the difference between a quantity of interest (outcome) and a suitable reference value.

• **Precision** refers to the consistency or repeatability of outcomes under the same conditions. Precision is the inverse of **noise**, also denoted **random error** (see Figure 1).

**Figure 1:**
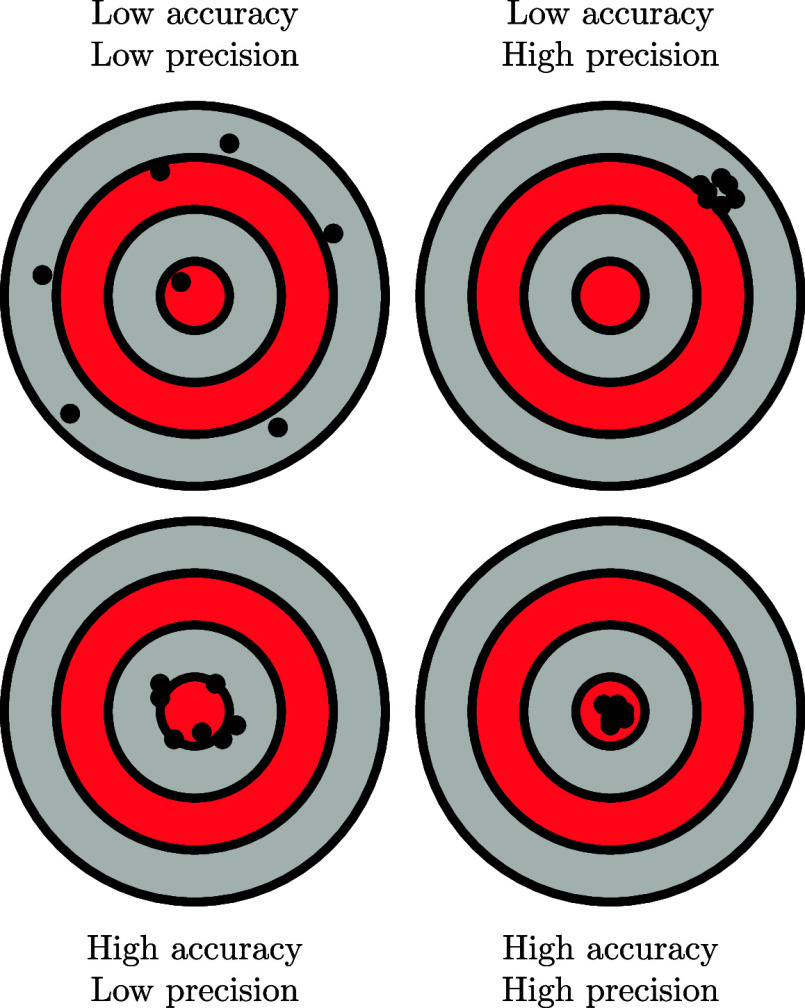
Illustration of accuracy and precision by black dots on gray/red targets.

• **Accuracy** describes how close an outcome is to its reference value. Accuracy is the inverse of **bias**, also denoted **systematic error** (see Figure 1).

• **Uncertainty** denotes the expected deviation of the outcome from a suitable reference value, prior to any comparison. While an error is the deviation of one individual calculated quantity of interest from the reference value, uncertainty refers to the expected distribution of the (unknown) error of an individual calculated quantity. This distribution can be made tangible by various quantities, such as, e.g., its standard deviation or a confidence interval.

• **Epistemic uncertainty** arises from a lack of knowledge about the chemical system or the computational model. It can, in principle, be reduced through improved models or more precise parametrizations.

• **Aleatoric uncertainty** is the part of overall uncertainty that cannot be reduced by more extensive sampling or by collecting more data. In computational chemistry, aleatoric uncertainty typically reflects variability in experimental reference data or stochasticity in simulations.

• **Uncertainty quantification** (UQ) refers to the systematic assessment and documentation of uncertainty in computational results. It encompasses the identification of uncertainty sources (see next section), their quantification, and their impact on the outcome.

• **Benchmarking** refers to the comparison of computational methods against reference data or other methods using predefined metrics. Unlike UQ, benchmarking assesses global performance rather than the reliability of individual predictions.^20,92–94^

• Errors are **deterministic** if they do not change when a calculation is repeated with the exact same input data.

• Errors are **stochastic** if they change randomly upon repeating a calculation with the exact same input data.

These definitions serve as the conceptual basis for discussing uncertainties with regard to *in silico* chemistry.

### 2.2 Sources of Uncertainty in *In Silico* Chemistry

Generally speaking, computational chemistry targets some *quantity of interest* (QoI). The *reference value f*(ξ) is a function of the chemical system ξ and can (at least in principle) be measured in an experiment. This can, for instance, be a reaction energy, a bulk property such as the density or the diffusion coefficient, or an infrared (IR) spectrum.

One then employs the computer implementation of some suitable model *f* ^comp^(ξ_chem_, η, *h*), which will depend on parameters describing (a model of) the chemical system ξ_chem_ as well as additional parameters η and *h* of the model and its numerical approximations, respectively, to calculate this QoI. The total error of this model is then given by

1 $$e_{\mathrm{tot}}=f^{\mathrm{comp}}\left(\xi_{\mathrm{chem}},\eta ,h\right)-f\left(\xi \right)$$

Uncertainty quantification aims at the systematic assessment and documentation of this error, i.e., the deviation of the predictions *for a specific chemical system* from the (usually unknown) chemical reality or (as proxy) an experimental or computational benchmark. It encompasses the identification of uncertainty sources (see next section), their quantification, and their impact on the outcome.

The uncertainty is introduced by many different sources. Their relative importance, and whether they are classified as aleatoric or epistemic, will depend both on the specific problem at hand (i.e., the quantity of interest in the computations and the benchmark that is targeted) and on the computational methods that are used.

As different sources of uncertainty will generally require different approaches for their quantification, it will be important to specify which sources of uncertainty are included in a specific method for uncertainty quantification in computational chemistry and to acknowledge sources of uncertainty that are not addressed. Neglecting some sources of uncertainty will generally correspond to redefining the benchmark that is used for comparison.

While many sources of uncertainty in computational chemistry have been discussed in the literature, there is currently no generally accepted classification scheme. Here, we will build on the categorization employed by Lejaeghere,^66^ who distinguishes *representation uncertainties*, *level of theory uncertainties*, and *numerics uncertainties* (Figure 2). In all three classes, uncertainties can be both deterministic and stochastic.

**Figure 2:**
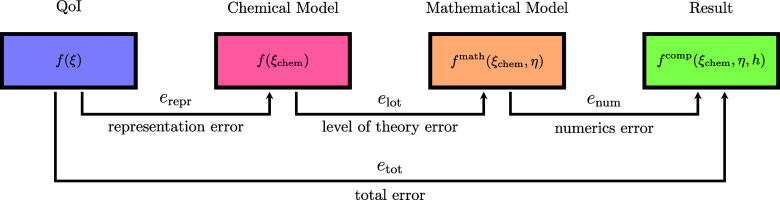
Illustration of the models (in colored boxes) and errors (denoted by arrows connecting the models) typically introduced during *in silico* chemistry workflows (also refer to Tables 1 and 2).

#### Representation Uncertainties

Lejaeghere defines these as follows:^66^ “This broadest class of errors represents all deviations between theory and reality because of deliberate approximations or inadvertent assumptions [that are not directly linked to the level of theory]. They correspond to a mismatch between what is calculated and what is experimentally measured, i.e., an incomplete atomistic representation of the conditions of the macroscopic material under study.”

First, this includes simplifications that are necessary to tackle a chemical problem computationally, which will lead to inadvertent mismatch between experiment and computation, i.e., real chemical systems are much more complicated than any computational model. In calculations for solids, this might include the neglect of impurities, disorder or microstructure. Similarly, when modeling solution chemistry side products or other impurities might be neglected. Boundary conditions usually cannot be represented faithfully in computational models, and one instead uses, e.g., periodic boundary conditions instead of finite-size bulk system, or an isolated molecule in vacuum instead of a gas-phase system at finite pressure.

Second, there are deliberate simplifications and approximations. Often, complex chemical systems are computationally not directly accessible, but simplified molecular models (e.g., using simplified ligands in a transition metal complex) have to be used instead. Similarly, it is common to either neglect the chemical environment of the chosen molecular model or to treat it using approximate models (e.g., a continuum solvation model). This can both be a deliberate approximation or a necessary simplification because of a lack of knowledge on the atomistic structure of the environment.

Altogether, such approximations define a chemical model ξ_chem_, and we can define the representation error as

2 $$e_{\mathrm{repr}}=f\left(\xi \right)-f\left(\xi_{\mathrm{chem}}\right)$$

#### Level of Theory Uncertainties

These are related to the approximations that are introduced in the computational chemistry methods that are used,^66^ which give rise to a well-defined mathematical model.^99^ This mathematical model *f* ^math^(ξ_chem_, η) will (explicitly or implicitly) depend on additional parameters η.

For quantum-chemistry, sources of such level of theory uncertainties include: the use of the Born–Oppenheimer approximation and a neglect of adiabatic corrections, the use of the nonrelativistic Schrödinger equation (i.e., neglect of relativistic and QED effects), the use of a simplified ansatz for the electronic wavefunction or the use of an approximate exchange–correlation functional in density-functional theory, the use of pseudopotentials for the core electrons, and the truncation of the one-electron basis set. For theoretical spectroscopy, further approximations in the respective methodology for calculating spectra (such as, e.g., the use of the harmonic approximation in the calculation of vibrational spectra) will introduce uncertainties.

For molecular dynamics simulations,^100,101^ sources of such uncertainties include the neglect of nuclear quantum effects in the Born–Oppenheimer approximation, the model that is assumed for the potential energy surface (e.g., the force field that is used), and the time scales of the simulation.

The approximations within the mathematical model lead to the level of theory error,

3 $$e_{\mathrm{lot}}=f\left(\xi_{\mathrm{chem}}\right)-f^{\mathrm{math}}\left(\xi_{\mathrm{chem}},\eta \right)$$

#### Numerics Uncertainties

The computer implementation of the mathematical model provides the final output of a computation, *f* ^comp^(ξ_chem_, η, *h*) and will depend on additional parameters *h*. Lejaeghere defines numerics uncertainties as follows:^66^ “Numerical errors [...] originate in the technical parts of a [DFT] calculation and vary from purely hardware- or algorithm-related effects, such as floating-point precision, to more physical approximations to reduce the computational load, such as series truncations or the use of a particular pseudopotential.”

For the case of solid-state DFT calculations using periodic boundary conditions, Herbst et al.^99^ list discretization error (such as Brillouin zone sampling, finite basis set of plane waves), algorithmic error (e.g., self-consistent Kohn–Sham equations are solved by a self-consistent field (SCF) algorithm, in which at each step of the SCF algorithm, a linear eigenvalue equation is solved with an iterative eigensolver), and arithmetic error (due to finite floating-point precision). They also point out that “to these must be added programming errors, not neglected in codebases consisting of millions of lines of code, and hardware errors, which are expected to become significant for exascale architectures”. For molecular dynamics simulations, the choice of a finite time step and cutoffs in the evaluation of the force field as well as the integration of the equations of motion itself contribute to numerics uncertainties.

The numerics error can be expressed as

4 $$e_{\mathrm{num}}=f^{\mathrm{math}}\left(\xi_{\mathrm{chem}},\eta \right)-f^{\mathrm{comp}}\left(\xi_{\mathrm{chem}},\eta ,h\right)$$

#### Discussion

The different models defined by Lejaeghere as discussed above are presented in Table 1, which also includes examples of corresponding approximations in computational chemistry. The errors introduced in the different steps are summarized in Table 2. Using the introduced notation and the triangle inequality, the magnitude of the total error can be decomposed into the error made by each step as follows:

$$\underset{\mathrm{total}\;\mathrm{error}}{\underset{︸}{\left|f\left(\xi \right)-f^{\mathrm{comp}}(\xi_{\mathrm{chem}},\eta ,h)\right|}}\leq \underset{\mathrm{representation error}}{\underset{︸}{\left|f\left(\xi \right)-f\left(\xi_{\mathrm{chem}}\right)\right|}}+\underset{\mathrm{level of theory error}}{\underset{︸}{\left|f\left(\xi_{\mathrm{chem}}\right)-f^{\mathrm{math}}\left(\xi_{\mathrm{chem}},\eta \right)\right|}}+\underset{\mathrm{numerics error}}{\underset{︸}{\left|f^{\mathrm{math}}\left(\xi_{\mathrm{chem}},\eta \right)-f^{\mathrm{comp}}\left(\xi_{\mathrm{chem}},\eta ,h\right)\right|}}$$

Often, the errors of the different steps can be investigated separately.

**Table 1:** Functions Describing the Sources of Uncertainty Introduced in a Typical Computational Chemistry Workflow, as Discussed in Section 2.2, Including Descriptions and Examples from Quantum Chemistry (QC) and Molecular Dynamics Simulations (MD) Perspectives^a^

function of model	description	example
*f*(ξ)	The *QoI* as a function of the underlying chemical system ξ.	QC: electronic energy, vibrational spectrum, reaction barrier; MD: density, diffusion coefficient, spectrum, (thermodynamic and transport properties), free energy.
*f*(ξ_chem_)	*Chemical model. ξ*_chem_ denotes the description of molecules (atomic resolution, in- or excluding electrons, coarse graining), structure (*xyz* coordinates of molecules), amount (single molecule, cluster, ensemble), and dynamical aspects (conformation, propagation in time).	Isolated molecule for electronic energy, molecule dissolved in liquid for explicit solvation, transition state of reaction for determining barrier, many molecules of a liquid simulated for several ns to determine diffusion coefficient, many molecules in crystal or surface, interface between many liquid molecules and solid molecules.
*f* ^math^(ξ_chem_, η)	*Mathematical model*. Everything that can be expressed in a mathematical notation and that is related to the computational chemistry method; may depend on additional parameters η.	Electronic-structure method (HF, post-HF, DFT), including LCAO ansatz, force field (atomistic, polarizable, coarse grained), equation of motion (velocity Verlet, leapfrog), equations for thermostats or barostats.
*f* ^comp^(ξ_chem_, η, *h*)	*Result* of the computer implementation of *f* ^math^(ξ_chem_, η), including parametrizations, mesh width, tolerances of solvers, etc., encoded in *h*.	Electronic energy at a chosen basis set, trajectory (includes coordinates and velocities at certain time steps for a certain duration of time), postprocession of the trajectory, free energy.

a For abbreviations used, refer to textbooks on QC^95^ or MD.^96–98^ .

**Table 2:** Compilation of Errors Occurring with *In Silico* Chemistry Workflows and Their Relation to Different Models^a^

representation error	*e*_repr_ = *f*(ξ) – *f*(ξ_chem_)
level of theory error	*e*_lot_ = *f*(ξ_chem_) – *f* ^math^(ξ_chem_, η)
numerics error	*e*_num_ = *f* ^math^(ξ_chem_, η) – *f* ^comp^(ξ_chem_, η, *h*)
**total error**	*e*_tot_ = *f*(ξ) – *f* ^comp^(ξ_chem_, η, *h*)

a Also refer to Table 1 and Figure 2.

However, the classification of different types of uncertainty is often not straightforward in computational chemistry. For instance, while the truncation of the one-electron basis set in molecular quantum chemistry (using atom-centered basis functions) is best classified as level-of-theory uncertainty, the truncation of a plane-wave basis set in condensed phase DFT calculations is governed by a single cutoff and can thus be classified as a source of numerics uncertainty.

Moreover, a core challenge in UQ for chemical computations is the lack of a clear hierarchy among sources of error. In the above classification, it is implicitly assumed that a computational model is built in a sequential manner: First, a structural model is chosen (e.g., positions of point-like nuclei), then a mathematical model (e.g., a specific level of approximation), and finally, numerical parameters are set. However, this linear view can be misleading. Theoretical assumptions often constrain which structural models are even meaningful or valid. Point-like nuclei, for example, are not merely a convenience but a precondition for the analytical integration of Coulomb operators. This feedback loop blurs the boundaries between different sources of error and complicates efforts to assign uncertainties in a strictly hierarchical fashion.

To navigate this complexity, it is often useful to distinguish between the modeling workflow and the classification of error sources. While the scheme proposed by Lejaeghere^66^—representation, level of theory, numerics—maps well onto the typical structure of computational models and was therefore used here to introduce key uncertainty concepts, it does not fully resolve the hierarchy problem.

An alternative taxonomy, put forward by Pernot,^67^ offers a more technically appropriate framework for analyzing uncertainty propagation and assessing the potential for uncertainty reduction. He distinguishes *model uncertainty*, *parameter uncertainty*, and *numerical uncertainty*.

Using the terminology introduced above, model uncertainty refers to uncertainties due to both the chemical model ξ_chem_ and the functional form of the mathematical model *f* ^math^. Uncertainties due to the chemical or mathematical model are almost always present in *in silico* chemistry. Their estimation is usually infeasible due to a lack of understanding of the property of interest *f*(ξ) and/or it requires a significant amount of domain specific knowledge. The best available option to quantify such uncertainties seems to be to compare models between each other or to consider a presumably highly precise or almost accurate model as the ground truth. Model uncertainties are systematic.

Uncertainties in the parameters of the mathematical model η, for example due to measurement errors and limited data availability, propagate through the model and affect its outcome. Since the precise error is unknown or *uncertain*, it is often modeled as random. Measurement errors are thus usually statistical errors and as such, can be quantified by statistical methods.

Numerical uncertainty comprises all uncertainties caused by algorithms for implementing the mathematical model of the target system and during runtime of the calculations. This includes round-off errors during linear algebra computations and tolerances *h* in approximate solvers. Computational errors are mostly systematic errors when computations are done on a single processor. Computations performed on parallel architectures are well-known for implying a stochastic aspect in the outcomes, particularly when many processors are involved.

The two discussed schemes for the classification of sources of uncertainty by Lejaeghere^66^ and Pernot^67^ share the distinction of *numerical uncertainties*. For the remaining sources of uncertainty, they employ complementary classifications. Pernot’s approach assumes a well-defined mathematical model that depends on uncertain parameters to distinguish *model uncertainty* and *parameter uncertainty*. While this might be an appropriate picture in some cases (such as regression models or more general ML-models with a well-defined reference), general modeling problems in *in silico* chemistry are often more complex and do not allow for a clear distinction of the computational model and its parameters. The scheme by Lejaeghere attempts to tame this complexity by distinguishing a chemical model (*representation uncertainty*) and a mathematical model (*level of theory uncertainty*). Both schemes are complementary, but neither seems to be perfectly suited to discuss all sources of uncertainty (and methods for their quantification) in *in silico* chemistry.

### 2.3 Uncertainty Quantification Workflow

Given a target quantity of interest *f*(ξ) and a computational model *f* ^comp^(ξ_chem_, η, *h*), an outline of an uncertainty quantification workflow could be sketched as follows:

1. Analyze the sources of uncertainty in the parameters of the computational model as outlined in section 2.2.

2. Analyze the sources of uncertainty in the parameters to determine whether they are deterministic or stochastic.

3. Understand and bound deterministic errors.

4. Develop a prior stochastic model for the stochastic errors.

5. If benchmarks (reference values) are available:

(a) Repeat steps items 1 to 4 for the benchmark.
(b) Use benchmark to update the prior model for the stochastic errors by means of an algorithm.

6. Compute desired statistical quantities of interest by means of an algorithm.

While items 1 and 2 can usually be answered by looking into the design of the computational model, the remaining three steps are quite challenging, but for different reasons. Items 3 and 4 are challenging from a theoretical perspective. As outlined in section 2.2, the error in the output data of a computational chemistry method can be bounded by the chemical and mathematical modeling errors, the computational error, and the measurement error. Modeling and computational errors are often considered as deterministic errors, but the complexity of the underlying models makes them difficult to understand even for domain experts. Measurement errors are usually modeled as stochastic error and can be quantified by computing various statistical quantities of interest for which a stochastic model is required. Developing stochastic models for measurement errors and errors in benchmarks is a topic on its own, and we refer to the relevant literature.^102,103^ On the other hand, the challenge of items 5 and 6 is mainly due to computational complexity, as computational methods in uncertainty quantification require many evaluations of computational chemistry methods, which are notoriously compute intensive. Two main problem classes can be distinguished, which we review in the following.

## 3 Theory of Uncertainty Quantification

### 3.1 Modeling Uncertainties in Computations

#### 3.1.1 A Crash Course in Probability Theory and Bayes’ Formula

The input data subject to stochastic uncertainty can be considered as a *random experiment*. A random experiment is associated with^104^

• the *outcomes* of the experiment, also referred to as *samples* or *realizations*;

• the *sample space*, that is, a set Ω consisting of all conceptually possible outcomes;

• the *events*, being subsets of Ω for which probability is defined.

A canonical example of a random experiment is the toss of a coin, where Ω = {heads, tails}. However, in the framework of computational chemistry, we can also think of the sample space as a set of possible molecule configurations or a set of functions describing a charge density.

To each event *A* ⊂ Ω we can assign[Fn fn1] a value *P*(*A*) ∈ [0, 1]. We say that *P* is a *probability function*, if *P*(Ω) = 1 and

5 $$P\left(\overset{\infty }{\underset{i=1}{\cup }}A_{i}\right)=\overset{\infty }{\underset{i=1}{\sum }}P\left(A_{i}\right)$$

for mutually disjoint sets *A*_*i*_ ∈ Ω which do not need to form a partition of Ω. From this definition, the basic rules of calculus for probabilities can be derived.^51,104^ For example, it is impossible that no event from the sample space occurs, i.e., *P*(Ø) = 0, the probability of the *opposite* of *A* ⊂ Ω is *P*(Ω \ *A*) = 1 – *P*(*A*), and the sum of probabilities to two not necessarily disjoint events *A*, *B* ∈ Σ can be expressed as

6 P(A)+P(B)=P(A∪B)+P(A∩B)

and eq 5 also holds for a finite number of sets. Here and in the following we write *A* ∪ *B* for the union and *A* ∩ *B* for the intersection of the sets *A* and *B* (illustrated in Figure 3).

**Figure 3:**
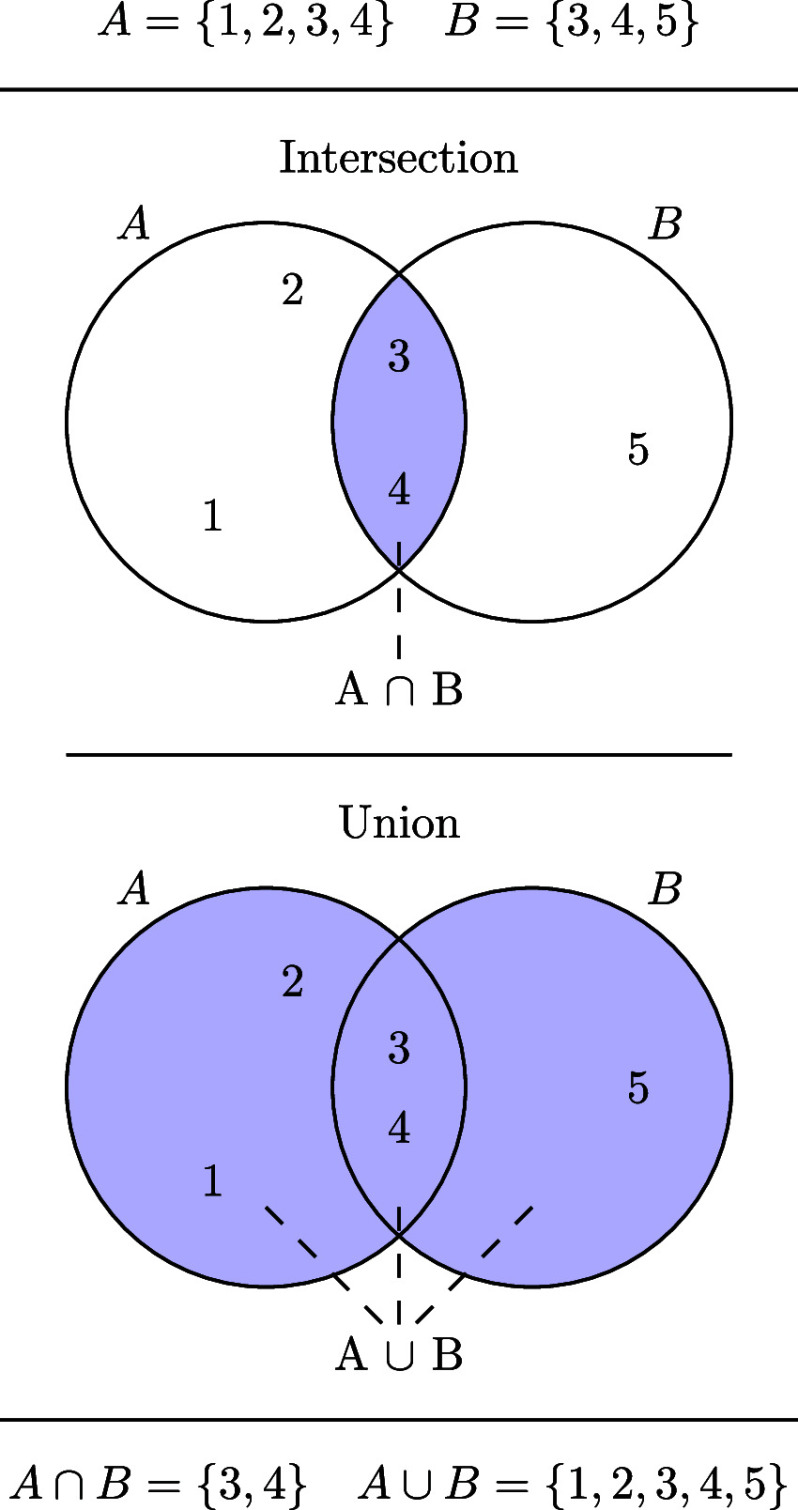
Illustration of the intersection (top) and union (bottom) of two sets *A* and *B*.

Given two events *A*, *B* ⊂ Ω with *P*(*B*) > 0, the *conditional probability of A given B* can be expressed as

7 $$P\left(A|B\right)=\frac{P\left(A\cap B\right)}{P\left(B\right)}$$

For *P*(*A*) = 0 we define *P*(*A*|*B*) = *P*(*A*). Using *Bayes’ formula*,^105^ *P*(*A*|*B*) can be expressed through

8 $$P\left(A|B\right)=\frac{P\left(B|A\right)P\left(A\right)}{P\left(B\right)}$$

In this formula, *P*(*A*|*B*) is also termed the *posterior*, *P*(*B*|*A*) is termed the *likelihood* of *A* given *B*, *P*(*A*) is termed the *prior*, and *P*(*B*) is termed the *marginal* probability. This formula is of particular importance when reference data and benchmarks need to be incorporated in inverse uncertainty quantification. Finally, we say that two events *A*, *B* ⊂ Ω with *P*(*A*), *P*(*B*) > 0 are *(stochastically) independent* if

9 P(A|B)=P(A),⁣orequivalently,⁣P(B|A)=P(B)

#### 3.1.2 Statistical Models and Random Variables

The quantities in computational chemistry which are affected by stochastic uncertainty can be considered as *random variables*. For the sake of simplicity in the presentation,[Fn fn2] we assume in the following that $\Omega \subset R^{d}$ , i.e., the sample space of the input parameters consists of vectors in $R^{d}$ , where *d* is the dimension. This can be motivated by the fact that most uncertainties in the input data of computational chemistry methods can be parametrized by a finite collection of real numbers. A *random variable* is a function *g*(ω), ω ∈ Ω, for which there exists a *probability distribution* ρ: Ω → [0, *∞*) such that

10 $$P\left(\left\{g\left(\omega \right):\omega \in A\right\}\right)=\int_{A}\rho \left(\omega \right)\;d\omega$$

It is this probability distribution which encodes most of the properties of the random variable. It follows from the definition that probability densities must satisfy

11 $$\int_{\Omega }\rho \left(\omega \right)\;d\omega =1$$

Similar properties as for probabilities can be derived. For example, we say that two random variables *x*_1_ and *x*_2_ with probability distributions ρ_1_ and ρ_2_ are *(stochastically) independent*, if their joint probability distribution is given by ρ­(ω_1_, ω_2_) = ρ_1_(ω_1_)­ρ_2_(ω_2_). Moreover, conditional probability distributions and Bayes’ formula can be derived, which reads

12 $$\rho \left(\omega |\omega^{\prime }\right)=\frac{\rho \left(\omega^{\prime }|\omega \right)\rho \left(\omega \right)}{\rho \left(\omega^{\prime }\right)}$$

A canonical example of a probability distribution is the *normal* or *Gaussian distribution* for real numbers x∈R :

13 $$\rho \left(x\right)=\frac{1}{\sqrt{2\pi }\sigma }\;\exp\left[-\frac{{\left(x-\mu \right)}^{2}}{2\sigma^{2}}\right]$$

where μ∈R and σ > 0. We write X∼N(μ,σ) for any random variable with this distribution. However, it is important to note that the concepts we discuss in the following are not restricted to Gaussian distributions, unless explicitly stated.

We note that the values of a random variable can be quite general, covering real values, molecule configurations, or functions describing charge distributions, for example. In the setting of computational chemistry, the results of the computational chemistry methods *g* = *f* ^comp^(ξ_chem_, η, *h*) or the outcome of a benchmark experiment *g* = *f* ^bench^(ξ) are canonical examples for random variables. The collection of a random variable with its probability distribution is also referred to as a *statistical model*.

#### 3.1.3 Statistical Quantities of Interest

The exact behavior of a random variable is often too complicated to be of interest in an uncertainty quantification analysis. Instead, different measures are usually considered. Most importantly, the *mean* of a random variable *g* is defined as

14 $$E\left[g\right]=\int_{\Omega }g\left(\omega \right)\rho \left(\omega \right)\;d\omega$$

and the *variance* and *standard deviation* are defined as

15 $$V\left[g\right]=E\left[g^{2}\right]-E{\left[g\right]}^{2},,\sigma \left[g\right]=\sqrt{V\left[g\right]}$$

Moreover, the *correlation* and *covariance* of *g* and some second random variable *h* are defined as

16 Cor[g,h]=E[gh]

17 Cov[g,h]=Cor[g,h]−E[g]E[h]

We write Cor­[*g*] = Cor­[*g*, *g*] and observe that V[g] = Cov­[*g*, *g*].

While a common understanding of random variables is that they should be real-valued, we mention for the sake of completeness that these quantities are also well-defined when *g* and *h* are function-valued. That is, they are real- or complex-valued functions *g*(*x*, ω), *h*(*x*, ω), depending on random events ω ∈ Ω and some additional parameter *x*. In this case, E[g] , V[g] , and σ­[*g*] are functions of *x* as well. The correlation Cor­[*g*, *h*] and covariance Cov­[*g*, *h*] become functions in two variables, defined through

$$\mathrm{Cor}\left[g,h\right]\left(x,y\right)=\int_{\Omega }g\left(x,\omega \right)h\left(y,\omega \right)\;d\omega$$

Cov[g,h](x,y)=Cor[g,h](x,y)−E[g](x)E[h](y)

For the even more general case when the product or integral are not defined, more advanced mathematical techniques can be used.^51^

Another measure to quantify the behavior of a random variable are *confidence intervals*, which express the probability that a random variable is within a certain range, i.e.,

18 P({a≤g≤b})≤ε

where 0 < ε < 1 and *a* and *b* are such that the comparison with *g* is reasonable (see Figure 4 for an illustration). A special case thereof is the *cumulative distribution function*

19 F(a)=P({a≤x})

which for real-valued random variables is the primitive of the probability density ρ.

**Figure 4:**
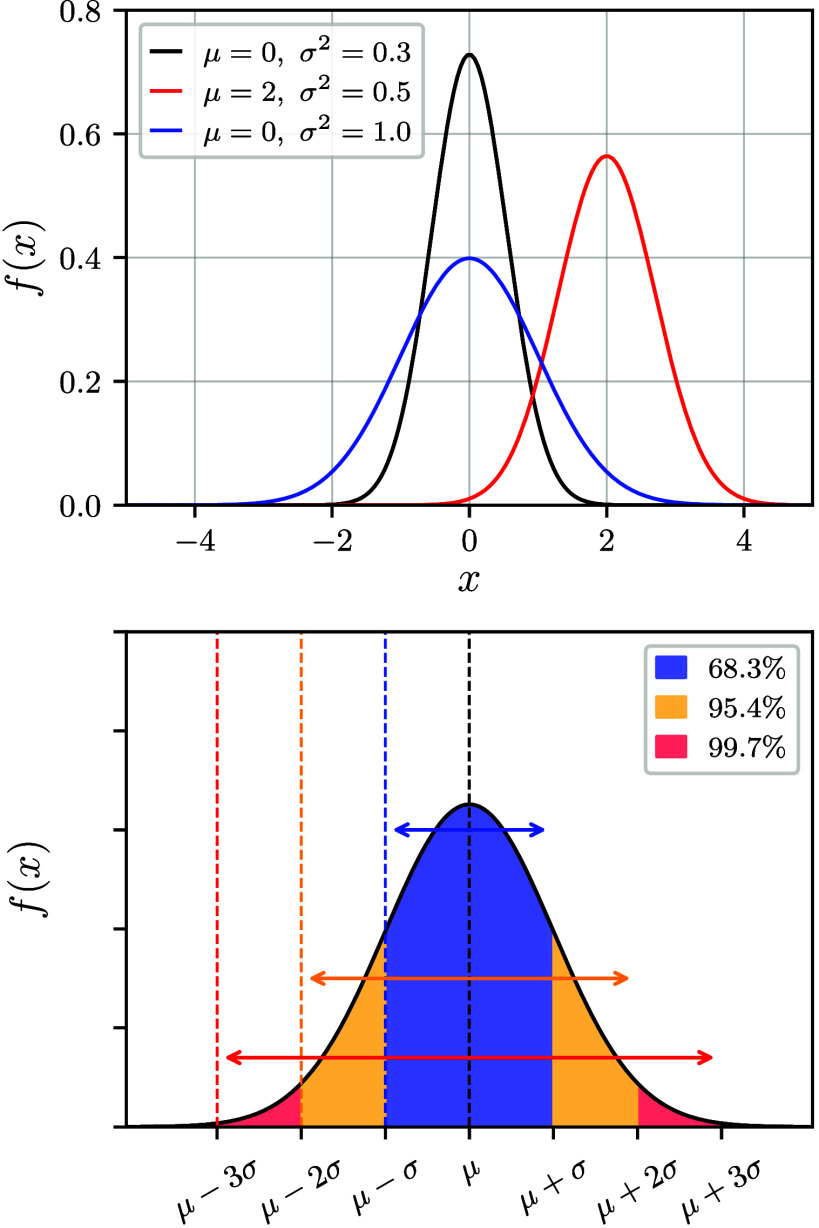
Illustration of normal (Gaussian) distributions. The top panel shows three distributions with different means and variances. The bottom panel illustrates the 1σ, 2σ, and 3σ confidence intervals.

Finally, *sensitivity indices* (cf. section 3.3) are measures that quantify how much a stochastic input parameter affects the outcome of a random model. As it turns out, it is quite common that the largest model variations are induced by only a few variables, with the remaining variables of reduced importance or negligible. The gained knowledge can be used for dimension reduction and the construction of surrogate models to obtain approximate but efficient computational models.^106^

### 3.2 Problem Classes of Uncertainty Quantification

Generally speaking, one can distinguish two problem classes of uncertainty quantification. In a *forward uncertainty quantification* problem (see section 3.2.1) one considers given or assumed uncertainties of the input data and model parameters, and investigates how these uncertainties propagates through the computational model. This mainly corresponds to items 3 and 4 of the UQ workflow sketched in section 2.3.

In an *inverse uncertainty quantification* problem, one takes benchmarks (reference data) into account and infers the uncertainties in the input data and/or model parameters by comparing these benchmarks to the output of the computational model. This mainly corresponds to item 5 of our general UQ workflow.

In both problem classes, some preliminary knowledge or assumptions on the behavior of the uncertainty are required. Most prominently, it needs to be decided whether the errors are modeled as *deterministic errors* or *stochastic errors* or both.

#### 3.2.1 Forward Uncertainty Quantification: Propagation of Uncertainty

Forward uncertainty quantification evaluates how errors in input data lead to errors in output data of a computational chemistry method. This is under the assumption that the input data is modeled as a *random variable* with known *probability distribution*. In the simplest case, when only the model parameters η are affected by stochastic uncertainties, we may model their uncertainty as

20 $$\eta =\eta_{0}+\nu$$

where ν is a random variable with known probability distribution ρ. This allows for the computation of statistical quantities of interest such as the mean,

21 $$E\left[f^{\mathrm{comp}}\left(\xi_{\mathrm{chem}},\eta ,h\right)\right]=\int f^{\mathrm{comp}}\left(\xi_{\mathrm{chem}},\eta ,h\right)\rho \left(\eta \right)\;d\eta$$

by replacing the integral by a numerical approximation based on evaluations of *f* ^comp^(ξ_chem_, η, *h*) for certain values of η. Note that η may lie in a high-dimensional parameter space and, thus, the numerical evaluation of the integral may suffer from the curse of dimensionality and prohibitive computational cost. We comment on appropriate numerical methods below.

#### 3.2.2 Inverse Uncertainty Quantification: Incorporating Benchmarks

Inverse uncertainty quantification problems most prominently occur in item 5 of our general UQ workflow (see section 2.3). Our starting point is a benchmark *f* ^bench^(ξ) (i.e., experimental or computational reference data for a certain input ξ). The benchmark can itself by subject to some uncertainty with respect to the true reference value *f*(ξ). For simplicity of presentation we assume in the following that the such uncertainties are negligible and refer to refs 51 and 107 for the more general case.

Given a benchmark *f* ^bench^(ξ), we perform a comparison to the prediction of the computational model,

22 $$f^{\mathrm{bench}}\left(\xi \right)-f^{\mathrm{comp}}\left(\xi_{\mathrm{chem}},\eta ,h\right)=\nu$$

and interpret this as a measurement subject additive Gaussian noise which is modeled as a realization of a random variable. Given a prior probability density ρ^prior^(η) for the model parameters η, our aim is to update this probability distribution by incorporating knowledge from the benchmark.

It then follows that ν is an N(0,Σ) -distributed random variable with probability distribution $\rho^{N}$ . From Bayes’ formula (eq 12), we obtain the *a* *posterior distribution* for η:

23 $$\rho^{\mathrm{post}}\left(\eta |f^{\mathrm{bench}}\right)=\frac{\rho^{N}\left(f^{\mathrm{bench}}-f^{\mathrm{comp}}\left(\xi_{\mathrm{chem}},\eta ,h\right)\right)\rho^{\mathrm{prior}}\left(\eta \right)}{Z}$$

where

24 $$Z=\int \rho^{N}\left(f^{\mathrm{bench}}-f^{\mathrm{comp}}\left(\xi_{\mathrm{chem}},\eta ,h\right)\right)\rho^{\mathrm{prior}}\left(\eta \right)\;d\eta$$

As for the forward problem, the posterior distribution can be used to compute various statistical quantities of interest, such as the mean:

25 $$E^{\mathrm{post}}\left[f^{\mathrm{comp}}\left(\xi_{\mathrm{chem}},\eta ,h\right)\right]=\int f^{\mathrm{comp}}\left(\xi_{\mathrm{chem}},\eta ,h\right)\rho^{\mathrm{post}}\left(\eta |f^{\mathrm{bench}}\right)d\eta$$

Here we need to point out that the integral of the normalization constant *Z* of the posterior distribution and for the posterior mean itself may be high-dimensional and thus compute intensive in practice. Further, we also note that the only difference between computing *Z* and the posterior mean is the presence of an additional factor *f* ^comp^(ξ, ζ, *h*) in the integrand.

### 3.3 Sensitivity Analysis

#### 3.3.1 Global Sensitivity Analysis: Identifying Influence of Parameters

Given a function in several variables, such as $f:{\left[0,1\right]}^{n}\to R$ , the idea of *sensitivity analysis* is to identify the variables which influence the result of *f* the most. The most popular tool to do so are *Sobol’ indices*,^108^ which explore that *f* can be decomposed into

26 $$f\left(x_{1},x_{2},...,x_{n}\right)=f_{0}+\underset{i_{1}}{\sum }f_{i_{1}}\left(x_{i_{1}}\right)+\underset{i_{1}i_{2}}{\sum }f_{i_{1}i_{2}}\left(x_{i_{1}},x_{i_{2}}\right)+...$$

with

27 $$f_{0}=\int f\left(x_{1},...,x_{n}\right)\;dx_{1}\cdot \cdot \cdot dx_{n}$$

28 $$f_{i_{1}}\left(x_{i_{1}}\right)=\int f\left(x_{1},...,x_{n}\right)\underset{k\neq i_{1}}{\prod }dx_{k}-f_{0}$$

29 $$f_{i_{1}i_{2}}\left(x_{i_{1}},x_{i_{2}}\right)=\int f\left(x_{1},...,x_{n}\right)\underset{k\neq i_{1},i_{2}}{\prod }dx_{k}-f_{0}-f_{i_{1}}\left(x_{i_{1}}\right)-f_{i_{2}}\left(x_{i_{2}}\right)$$

etc. It is intuitively clear that the terms in eq 26 provide some insight on how *f* depends on its various variables. For this reason, one also refers to eq 26 as an *analysis of variance, or similiarly, ANOVA decomposition*. One can show that the variances of the entities are given as

30 $$V\left[f\right]=\int f{\left(x\right)}^{2}\;dx-{f_{0}}^{2}$$

31 $$V\left[f_{i_{1}\cdot \cdot \cdot i_{s}}\right]=\int f_{i_{1}\cdot \cdot \cdot i_{s}}{\left(x_{i_{1}},...,x_{i_{s}}\right)}^{2}\;dx_{i_{1}}\cdot \cdot \cdot dx_{i_{s}}$$

and that it holds

32 $$V\left[f\right]=\overset{n}{\underset{s=1}{\sum }}\underset{i_{1}<\cdot \cdot \cdot <i_{s}}{\sum }V\left[f_{i_{1}\cdot \cdot \cdot i_{s}}\right]$$

The latter expression makes clear that the *global sensitivity indices* or *Sobol’ indices*

33 $$S_{i_{1}\cdot \cdot \cdot i_{s}}=\frac{V\left[f_{i_{1}\cdot \cdot \cdot i_{s}}\right]}{V\left[f\right]}$$

allow identification of the contributions to *f* which contribute the most to V[f] . Therein, *s* is called the *order* or *dimension* of the index. One can easily check that the indices are nonnegative and their sum is 1. The latter fact is particularly useful in practice, as one may only compute first order indices to begin with and can then increase the order until the sum of the indices is approximately one. Several other indices to quantify the impact of parameters exist and we refer to the relevant literature for an overview.^49,60,109^

#### 3.3.2 Local Sensitivity Analysis: Perturbation Based Uncertainty Quantification

While a global sensitivity analysis usually requires approaches requiring multiple or many evaluations of *f*(*x*), *local sensitivity analysis*,^49,109^ also called the *perturbation approach*,^110,111^ is well-suited for situations where the values of *x* are small random fluctuations of bounded magnitude *h*_0_ around a reference value *x*_0_, i.e.,

34 $$x=x_{0}+h,,\mathrm{with}\;h\;\mathrm{random}\;\mathrm{and}\;\left|h\right|<h_{0}$$

The randomness of the model outcomes can then be approximated as a Taylor expansion, i.e.,

35 $$f\left(x\right)=f\left(x_{0}+h\right)\approx f\left(x_{0}\right)+hf^{\prime }\left(x_{0}\right)+\frac{h^{2}}{2}f^{\prime \prime }\left(x_{0}\right)+...$$

Assuming some knowledge of the statistics of *h*, this allows for the approximation of statistical quantities of interest, such as the mean:

36 $$E\left[f\left(x\right)\right]\approx f\left(x_{0}\right)+E\left[h\right]f^{\prime }\left(x_{0}\right)+\frac{E\left[h^{2}\right]}{2}f^{\prime \prime }\left(x_{0}\right)+...$$

For small perturbations, i.e., small *h*_0_, these approximations are often more accurate than sampling-based approaches and can save orders of magnitude in compute time. The quantities E[h] , $E\left[h^{2}\right]$ , etc. can be computed in closed form or approximated by efficient numerical methods without the need for sampling.^110,112–115^ While local sensitivity analysis is traditionally only employed for forward problems, expansions for Bayesian inverse problems also exist.^116^

### 3.4 Sampling Methods

#### 3.4.1 Monte Carlo Methods

One of the central tasks when computing statistical quantities of interest (see section 3.1.3) such as the mean in eq 14 is to evaluate the corresponding integrals. In general, since the functions to integrate are usually not available analytically, these integrals need to be evaluated by numerical methods.

The most prominent and simplest approach is the *Monte Carlo estimator*.^53,97,117^ That is, given a random variable *f*(*x*) with probability distribution ρ­(*x*), the integral is approximated as an average

37 $$E\left[f\right]=\int_{\Omega }f\left(\omega \right)\rho \left(\omega \right)\;d\omega \approx \frac{1}{N}\overset{N}{\underset{i=1}{\sum }}f\left(\xi_{i}\right)≕E_{N}\left[f\right]$$

where *ξ*_*i*_, *i* = 1, ..., *N*, are identically distributed, stochastically independent (i.i.d. in short) random samples drawn from the distribution ρ. It is important to realize that the Monte Carlo estimator is a sum of random variables and, thus, a random variable itself, i.e., its outcome will change with every realization. To this end, it is well-known that the Monte Carlo estimator satisfies the following bound for the *root-mean-square error*:

38 $$\sqrt{E\left[{\left(E\left[f\right]-E_{N}\left[f\right]\right)}^{2}\right]}=\sqrt{\frac{V\left[f\right]}{N}}$$

and that for any ε > 0 it holds the *failure probability*

39 $$P\left(\left\{\left|E\left[f\right]-E_{N}\left[f\right]\right|>\varepsilon \right\}\right)\leq \frac{V\left[f\right]}{N\varepsilon^{2}}$$

The first estimate tells us that the root-mean-square error of the Monte Carlo method indeed converges to the true mean, with a *convergence rate* of $O\left(N^{-1/2}\right)$ . Unfortunately, this rate can be painstakingly slow in practice since in practice an evaluation of *f*(*x*_*i*_) will usually involve the run of a computational chemistry method, which can be quite expensive. For example, estimating the mean of *f* up to an accuracy of 10^–3^ in the root-mean-square error requires 10^6^ samples due to eq 37. However, the latter estimate tells us that, no matter how many samples we use in our Monte Carlo estimator, we can never exclude the possibility that the error of a single realization can be arbitrarily wrong, although with a probability which decreases with *N*.

This unsatisfactory behavior of the Monte Carlo estimator has led to the development of a vast amount of methods which promise to overcome its downsides, the most prominent of which we review below. However, let us note that in some cases simple *variance reduction* techniques^117^ allow for small modifications in the sampling process to lower the magnitude of V[f] and thus improve the error and the failure probability. This being said, the Monte Carlo estimator is still the simplest method to implement and all of the following methods require additional information on the problem at hand. In contrast, the only assumption made by the Monte Carlo method is that V[f] is finite and that one can sample from the probability distribution of *f*. The latter assumption can be weakened using *Markov chain Monte Carlo (MCMC)* methods,^118,119^ which do not require explicit access to the probability distribution. The development of MCMC methods is an active field of research, and over the years various popular developments such as adaptive MCMC,^120,121^ Hamiltonian Monte Carlo,^122,123^ and hybrid Monte Carlo^124^ have evolved.

#### 3.4.2 Resampling Schemes

In practice, it may happen that sampling from probability distribution ρ of a random variable *X* is not accessible due to theoretical, experimental, or computational constraints, but a finite number of samples $X=\left\{X_{1},...,X_{M}\right\}$ of *X* is available. For example, properties derived from quantum-chemical calculations are often both high-dimensional with respect to molecular structure and expensive to obtain, which implies that the underlying distribution ρ remains effectively unknown.

The idea of *resampling schemes* is to draw samples from the set X rather than sampling from ρ directly. This resampling procedure can then be used to compute statistical quantities of interest with the Monte Carlo method, for example. Depending on whether the samples are drawn with or without replacement and whether the set of drawn samples has smaller or the same cardinality, the arising estimators are termed the randomization test,^125–127^ the jackknife,^128,129^ or the bootstrap method.^130–132^ For a comprehensive comparison of the sampling schemes we refer the reader to ref 133 (see also Figure 5).

**Figure 5:**
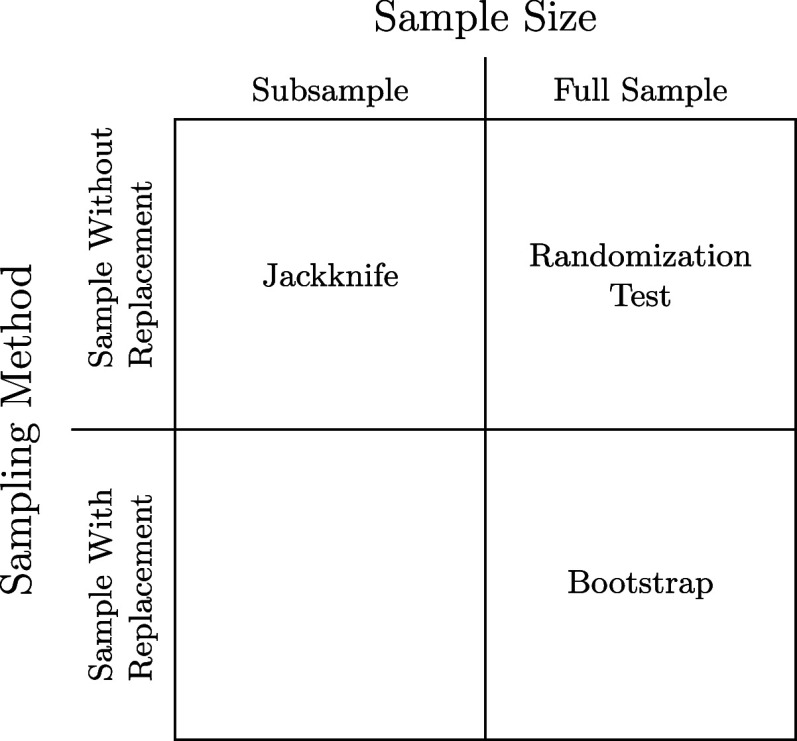
Comparison of resampling schemes. Reproduced with permission from ref 133. Copyright 1999 Lawrence Erlbaum Associates.

The simplicity of resampling methods has led to enormous popularity across the fields. On the other hand, it is intuitively clear that it heavily depends on how representative the samples in X are compared to *X*. Still, for certain cases one can show that the empirical cumulative distribution function of X converges in a reasonable sense to the one of *X* as *M* → *∞*, i.e., if we enlarge the size of our sample set to infinity. For an in-depth discussion and examples we refer to the respective references.

#### 3.4.3 Quadrature Methods: Quasi-Monte Carlo and Sparse Grids

*Quadrature methods* can be considered as a modification of the Monte Carlo estimator where the random samples are replaced by deterministic ones. To this end, one approximates

40 $$E\left[f\right]=\int_{\Omega }f\left(\omega \right)\rho \left(\omega \right)\;d\omega \approx \overset{N}{\underset{i=1}{\sum }}w_{i}f\left(\xi_{i}\right)≕Q\left[f\right]$$

where the *ξ*_*i*_, *i* = 1, ..., *N*, are deterministic *quadrature nodes* and the *w*_*i*_, *i* = 1, ..., *N*, are *quadrature weights*. As a straightforward modification of the Monte Carlo approach, *quasi-Monte Carlo methods* ^53,134^ choose the quadrature nodes *ξ*_*i*_, *i* = 1, ..., *N*, deterministically and the quadrature nodes as $w_{i}=\frac{1}{N}$ , *i* = 1, ..., *N*. The intuition behind this is that the Monte Carlo estimator can potentially become more accurate if the quadrature nodes are of low discrepancy (i.e., evenly distributed) but still chosen at reasonably sophisticated places.^134^ Indeed, classical quasi-Monte Carlo methods provide a convergence rate of almost 1, i.e., $O\left(N^{-1+\delta }\right)$ for any δ > 0, if first-order mixed regularity of the integrand is available.^56,134^ If the integrand provides even more regularity, even higher orders of convergence can be achieved when using modern quasi-Monte Carlo methods.^135–137^ Due to their deterministic choice of the quadrature nodes, quasi-Monte Carlo methods lead to a *deterministic approximation* of the integral, rather than a statistical estimator as the Monte Carlo Method.

Traditional deterministic quadrature rules leverage the freedom of also choosing the quadrature weights to obtain improved deterministic approximations of the integral. For single parameter problems, i.e., Ω⊂R , it is textbook knowledge that choosing quadrature nodes and quadrature weights according to *Gauss quadrature rules* yields the best possible approximation of the integral in eq 40. However, the situation is different when the domain of integration Ω is higher dimensional. Then, the naive combination of one-dimensional quadrature formulas by means of a tensor-product approach leads to methods suffering from the *curse of dimensionality*. To overcome the curse of dimensionality, the idea of *sparse grid methods* ^54,138^ is that a more sophisticated combination of one-dimensional quadrature formulas can break the curse of dimensionality, if the integrand is sufficiently smooth. More precisely, given a sequence of *nested one-dimensional quadratures* on Ω=I⊂R , i.e.,

41 $$Q_{l}\left[f\right]=\overset{N_{l}}{\underset{i=1}{\sum }}w_{l,i}f\left(\xi_{l,i}\right),,l=0,1,2,...$$

with $\left\{\xi_{l,1},...,\xi_{l,N_{l}}\right\}\subset \left\{\xi_{1,l+1},...,\xi_{N_{l+1},l+1}\right\}$ , l = 0, 1, 2, ..., we introduce the differences

42 $$\Delta_{l}\left[f\right]=Q_{l}\left[f\right]-Q_{l-1}\left[f\right],,l=0,1,2,...$$

with the convention that *Q*_–1_[*f*] = 0. We note that $\Delta_{l}\left[f\right]$ is similar to $Q_{l}\left[f\right]$ in the sense that $\Delta_{l}\left[f\right]$ is a quadrature rule with the same nodes but with different weights.

Whereas the tensor product quadrature rule for a *d*-dimensional domain $\Omega =\underset{d\;\mathrm{times}}{\underset{︸}{I\times \cdot \cdot \cdot \times I}}\subset R^{d}$ is given by application of *Q*_*l*_[·] in each variable, i.e.,

43 $$Q_{l}^{\left(d,\mathrm{TP}\right)}\left[f\right]=\underset{d\;\mathrm{times}}{\underset{︸}{\left(Q_{l}⊗\cdot \cdot \cdot ⊗Q_{l}\right)}}\left[f\right]=\overset{N_{l}}{\underset{i_{1}=1}{\sum }}\cdot \cdot \cdot \overset{N_{l}}{\underset{i_{d}=1}{\sum }}w_{i_{1}}\cdot \cdot \cdot w_{i_{d}}f\left(\xi_{i_{1}},...,\xi_{i_{d}}\right)$$

we can rearrange terms to rewrite it as

44 $$Q_{l}^{\left(d,\mathrm{TP}\right)}\left[f\right]=\underset{∥i{∥}_{\infty }\leq l}{\sum }\left(\Delta_{i_{1}}⊗\cdot \cdot \cdot ⊗\Delta_{i_{d}}\right)\left[f\right]$$

where $i=\left(i_{1},...,i_{d}\right)\subset N_{0}^{d}$ and ∥**i**∥_*∞*_ = max_*k*=1,...,*d*_|*i*_*k*_|. The sparse quadrature rule is then given as^138,139^

45 $$Q_{l}^{\left(d,\mathrm{SG}\right)}\left[f\right]=\underset{∥i{∥}_{1}\leq l+d-1}{\sum }\left(\Delta_{i_{1}}⊗\cdot \cdot \cdot ⊗\Delta_{i_{d}}\right)\left[f\right]$$

where $i=\left(i_{1},...,i_{d}\right)\subset N_{0}^{d}$ and ∥**i**∥_1_ = *∑*_*k*__=1,...,*d*_|*i*_*k*_|. Using the combination technique,^140^ the latter expression can be simplified to^139,141^

46 $$Q_{l}^{\left(d,\mathrm{SG}\right)}\left[f\right]=\underset{∥i{∥}_{1}\leq l+d-1}{\sum }{\left(-1\right)}^{l+d-∥i{∥}_{1}-1}\left(\begin{array}{c} d-1\\ {∥i∥}_{1}-l \end{array}\right)\cdot \left(Q_{i_{1}}⊗\cdot \cdot \cdot ⊗Q_{i_{d}}\right)\left[f\right]$$

One can show that this quadrature rule satisfies an error bound of $O\left(N^{-r}{\left(\log\;N\right)}^{\left(d-1\right)\left(r+1\right)}\right)$ , where *N* is the number of required evaluations of *f* if *f* provides *r*th-order mixed regularity.^142^ This rate of convergence can be improved, if one allows for different quadrature rules in the different variable directions which can be accomplished using a priori information^143,144^ or adaptively.^145^ We refer the interested reader to the relevant literature^54,139,146^ for more details and remarks and to Figure 6 for an illustration.

**Figure 6:**
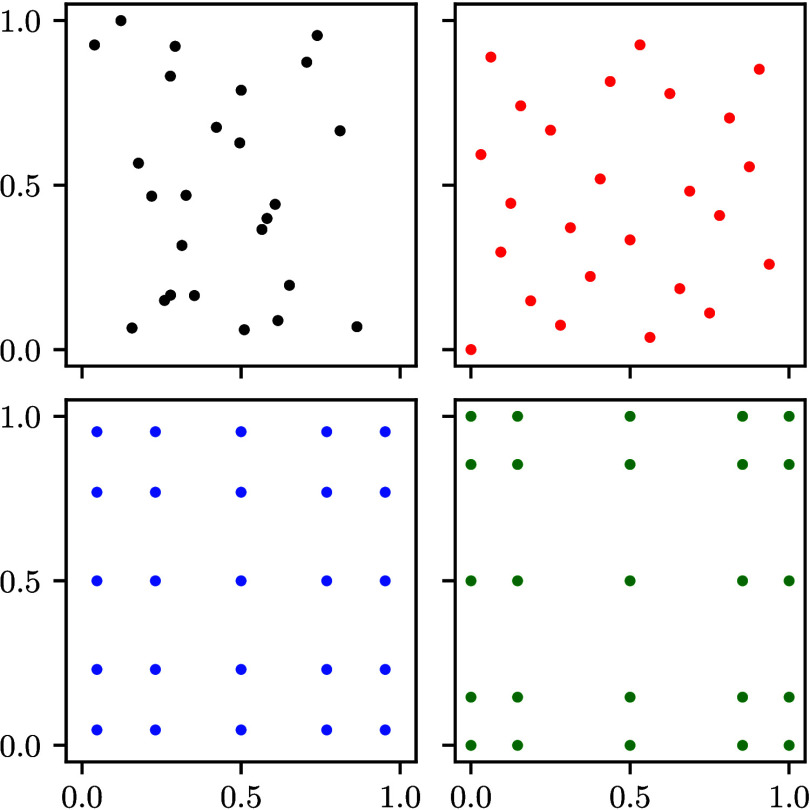
Illustration of different quadrature methods for 25 points in Ω = [0, 1]^2^. Top left (black): Random Monte Carlo points. Top right (red): Quasi-Monte Carlo points from a 2,3 Halton series. Bottom left (blue): Gaussian quadrature points approximating a uniform distribution. Bottom right (green): Clenshaw–Curtis quadrature points approximating a uniform distribution.

Fortunately, sparse grid techniques are available in most software packages for uncertainty quantification nowadays, see section 3.8, and the average user does not need to dive into the implementation details of sparse grids.

#### 3.4.4 Spectral Approaches: Stochastic Collocation and Polynomial Chaos

Spectral approaches^49,110^ belong to the class of surrogate models and are based on polynomial model approximations. They can be expected to work well if the model changes sufficiently smooth in the parameters. More precisely, for a model in *d* variables represented by a sufficiently smooth function $f:{\left[0,1\right]}^{d}\to R$ , one approximates

47a $$f\left(x\right)\approx \underset{i\in I}{\sum }c_{i}\Psi_{i}\left(x\right)$$

47b $$\Psi_{i}\left(x\right)=\Psi_{i_{1}}\left(x_{1}\right)\cdot \cdot \cdot \Psi_{i_{d}}\left(x_{d}\right)$$

where $I\subset N_{0}^{d}$ is a suitable (multi-index) set of polynomial degrees, **i** = (*i*_1_, ..., *i*_*d*_) is an element thereof, *c*_**i**_ are suitable coefficients, and ${\left\{\Psi_{l}\right\}}_{l}$ is a family of polynomials in one variable. The main challenge in using this approach is choosing a family of polynomials $\Psi_{l}$ and polynomial degrees (i.e., the index set I ) and then to determine the coefficients *c*_**i**_. The family of polynomials directly affects the way in which the coefficients can be determined, the approximation quality, and other properties. Similar to tensor product quadrature one could use different types of polynomials in each variable, but we keep this simple presentation for the sake of illustration.

We typically speak of *stochastic collocation* ^147^ methods if we choose to interpolate *f* at a series of predetermined points in the parameter space. A classical choice to do so is to choose a set of pairwise distinct interpolation points (ξ_0_, ..., *ξ*_*N*_) ⊂ [0, 1] and consider associated one-dimensional Lagrange polynomials

48 $$\Psi_{l}\left(t\right)=\overset{N}{\underset{\begin{array}{c} i=0\\ i\neq l \end{array}}{\prod }}\frac{t-\xi_{i}}{\xi_{l}-\xi_{i}},,l=0,...,N$$

Lagrange polynomials (visualized in Figure 7, top panel) have the convenient property that

49 $$\Psi_{l}\left(\xi_{k}\right)=\{\begin{array}{ll} 1, & l=k\\ 0, & l\neq k \end{array},k,l=0,1,...,N$$

which allows an approximation of the type shown in eq 47 to be obtained by setting

50 $$f\left(x\right)\approx \underset{∥i{∥}_{\infty }\leq N}{\sum }f\left(\xi_{i_{1}},...,\xi_{i_{d}}\right)\Psi_{i_{1}}\left(x_{1}\right)\cdot \cdot \cdot \Psi_{i_{d}}\left(x_{d}\right)$$

Similar to tensor product quadrature, this naive construction will suffer from the curse of dimensionality in the evaluation points. If the function to approximate is sufficiently smooth, sparse grid techniques can be used in complete analogy to the case of quadrature methods.^54,138,148,149^

**Figure 7:**
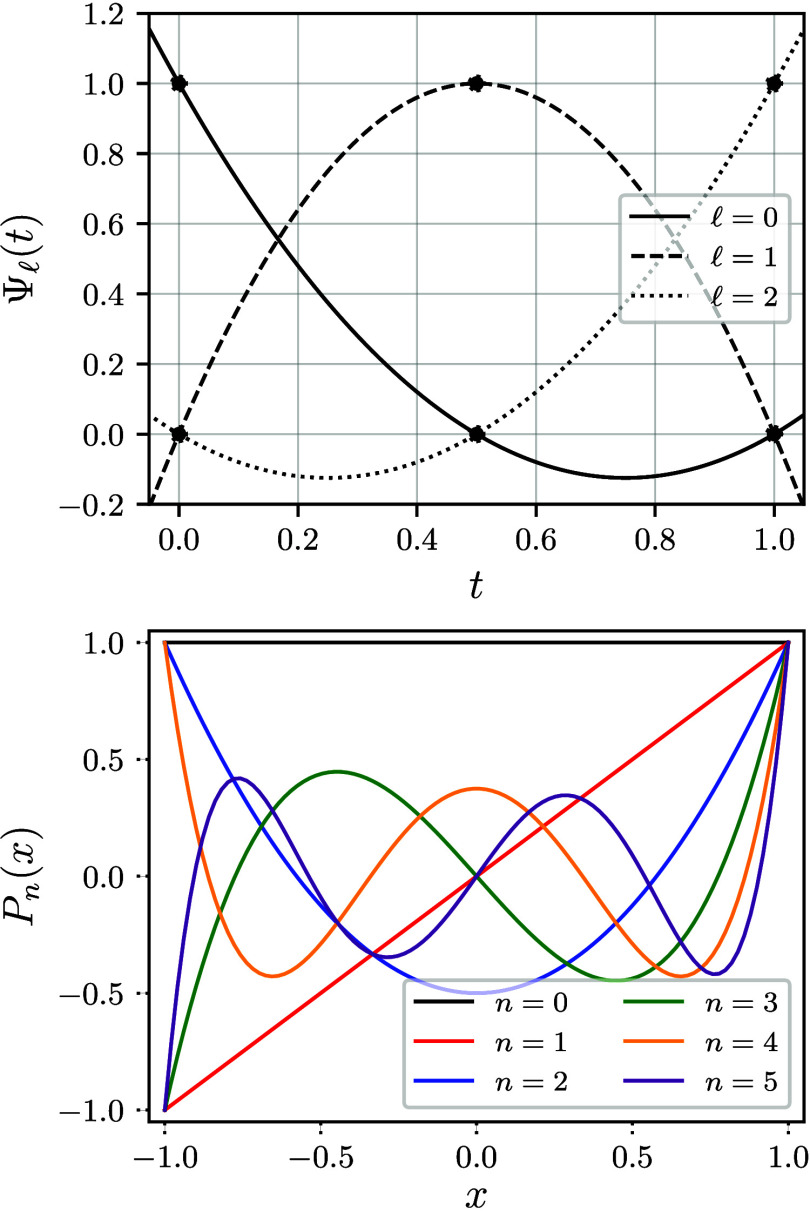
Illustration of three Lagrange polynomials of degree 2 for interpolation points ξ_0_ = 0, ξ_1_ = 0.5, ξ_2_ = 1, denoted by black lines and dots (top panel) and the first six Legendre polynomials (bottom panel).

In contrast to stochastic collocation methods, *polynomial chaos expansions*,^150–152^ classically choose *orthogonal polynomials*. Assuming that *f*(*x*) = *f*(*x*_1_, ..., *x*_*d*_) has finite variance and depends on i.i.d. random variables *x*_1_, ..., *x*_*d*_, each of them following the probability distribution ρ_1_, we introduce the joint probability distribution ρ­(*x*_1_, ..., *x*_*d*_) = ρ_1_(*x*_1_)···ρ_*d*_(*x*_*d*_) and an *inner product* and *norm*:

51 $${\left\langle f,g\right\rangle }_{\rho }=\int_{\Omega }f\left(x\right)g\left(x\right)\rho \left(x\right)\;dx$$

52 $$∥f{∥}_{\rho }=\sqrt{{\left\langle f,f\right\rangle }_{\rho }}=\sqrt{\int_{\Omega }f{\left(x\right)}^{2}\rho \left(x\right)\;dx}$$

Orthogonal polynomials are families of polynomials ${\left\{\Psi_{l}\right\}}_{l=0}^{\infty }$ which satisfy

53 $${\left\langle \Psi_{l},\Psi_{k}\right\rangle }_{\rho }=\{\begin{array}{ll} 1, & l=k\\ 0, & l\neq k \end{array},k,l=0,1,2,...$$

and each $\Psi_{l}$ is a polynomial of degree l . Typical examples for orthogonal polynomials are Hermite polynomials (with ρ_1_ being the standard normal distribution on R ) and Legendre polynomials (with ρ_1_ being the uniform distribution on [−1, 1]). Legendre polynomials are visualized in the bottom panel of Figure 7. Orthogonal polynomials exist for all probability distributions, but their computation is nontrivial except for very simple cases. Once the orthogonal polynomials are available, an approximation of the form eq 47 is given by

54 $$f\left(x\right)\approx \underset{∥i{∥}_{\infty }\leq N}{\sum }{\left\langle f,\Psi_{i_{1}}⊗\cdot \cdot \cdot ⊗\Psi_{i_{d}}\right\rangle }_{\rho }\Psi_{i_{1}}\left(x_{1}\right)\cdot \cdot \cdot \Psi_{i_{d}}\left(x_{d}\right)$$

We note that evaluating ${\left\langle f,\Psi_{i_{1}}⊗\cdot \cdot \cdot ⊗\Psi_{i_{d}}\right\rangle }_{\rho }$ requires the computation of an integral, which can for example be done with quadrature methods (cf. section 3.4.3) or a least-squares fit.^153^ Similar to stochastic collocation, our presentation can be modified to allow for different probability distributions in each random variable and the assumption of stochastic independence can be lifted.^154^ Moreover, the polynomial chaos expansions as introduced here will suffer from the curse of dimensionality which can be tacklet with sparse polynomial chaos expansions.^155,156^

Once the approximation polynomial is available, it can be used to compute statistical quantities of interest such as the mean, variance, or sensitivity measures,^157^ for example.

For a discussion on the advantages and disadvantages of the approaches, we refer the reader to ref 49.

#### 3.4.5 Multilevel and Multifidelity Methods

The idea of *multilevel* ^57,158^ *and multifidelity* ^58^ *methods* is to exploit (approximate) multilevel hierarchies to construct a variance reduction technique and shift a large amount of sampling costs from accurate but expensive models to less accurate but cheap models. The origin of these methods is in the uncertainty quantification of partial differential and integral equations, where different levels of model accuracy and computational cost can easily be constructed by nested hierarchies of meshes and/or approximation spaces. To this end, the classical multilevel Monte Carlo method considers a model *f* which is approximated by a sequence of approximations *f*_0_, *f*_1_, ..., *f*_*L*_ with increasing accuracy. In this way, the mean of the most accurate approximation $E\left[f_{L}\right]$ can be approximated by a telescopic sum:

55 $$E\left[f\right]\approx E\left[f_{L}\right]=E\left[f_{0}\right]+\overset{L}{\underset{l=1}{\sum }}E\left[f_{l}-f_{l-1}\right]\approx E_{N_{0}}\left[f_{0}\right]+\overset{L}{\underset{l=1}{\sum }}E_{N_{l}}\left[f_{l}-f_{l-1}\right]=E_{L}^{\mathrm{ML}}\left[f\right]$$

where the $N_{l}$ are the number of samples for the various Monte Carlo estimators. The latter approximation is called the *multilevel Monte Carlo estimator*, visualized in Figure 8. Now we write *f*_–1_ = 0 and let $C_{l}$ be the computational cost for the evaluation of one sample of $f_{l}-{f_{l}}_{-1}$ . Then the overall cost of the multilevel Monte Carlo estimator is $C=\overset{L}{\underset{l=0}{\sum }}N_{l}C_{l}$ , and its variance is^57^

56 $$V\left[E_{L}^{\mathrm{ML}}\left[f\right]\right]=\overset{L}{\underset{l=0}{\sum }}\frac{V\left[f_{l}-f_{l-1}\right]}{N_{l}}$$

The estimator will be accurate if this variance is small. One can show that for any desired upper bound for this variance, choosing $N_{l}∼\sqrt{V\left[f_{l}-f_{l-1}\right]/C_{l}}$ minimizes the overall computational cost of the estimator.^57^ Thus, if $V\left[f_{l}-f_{l-1}\right]$ decays sufficiently quickly with l , then we obtain small $V\left[E_{L}^{\mathrm{ML}}\left[f\right]\right]$ (i.e., an accurate estimator) with small sample numbers $N_{l}$ for computationally expensive models (large l ) and large sample numbers $N_{l}$ for computationally cheap models (small l ). Compared to the conventional (single-level) Monte Carlo method (cf. section 3.4.1), where all samples need to be drawn on the level with the highest accuracy (here: *L*) this can yield significant savings in compute time. Considerations with the same outcome can be made when replacing the single-level Monte Carlo estimators in the multilevel estimator by quadrature methods such as quasi-Monte Carlo, sparse grids, stochastic collocation, etc. In fact, the multilevel Monte Carlo method can itself be interpreted as a sparse grid method,^159^ giving rise to the family of multi-index Monte Carlo methods.^160^

**Figure 8:**
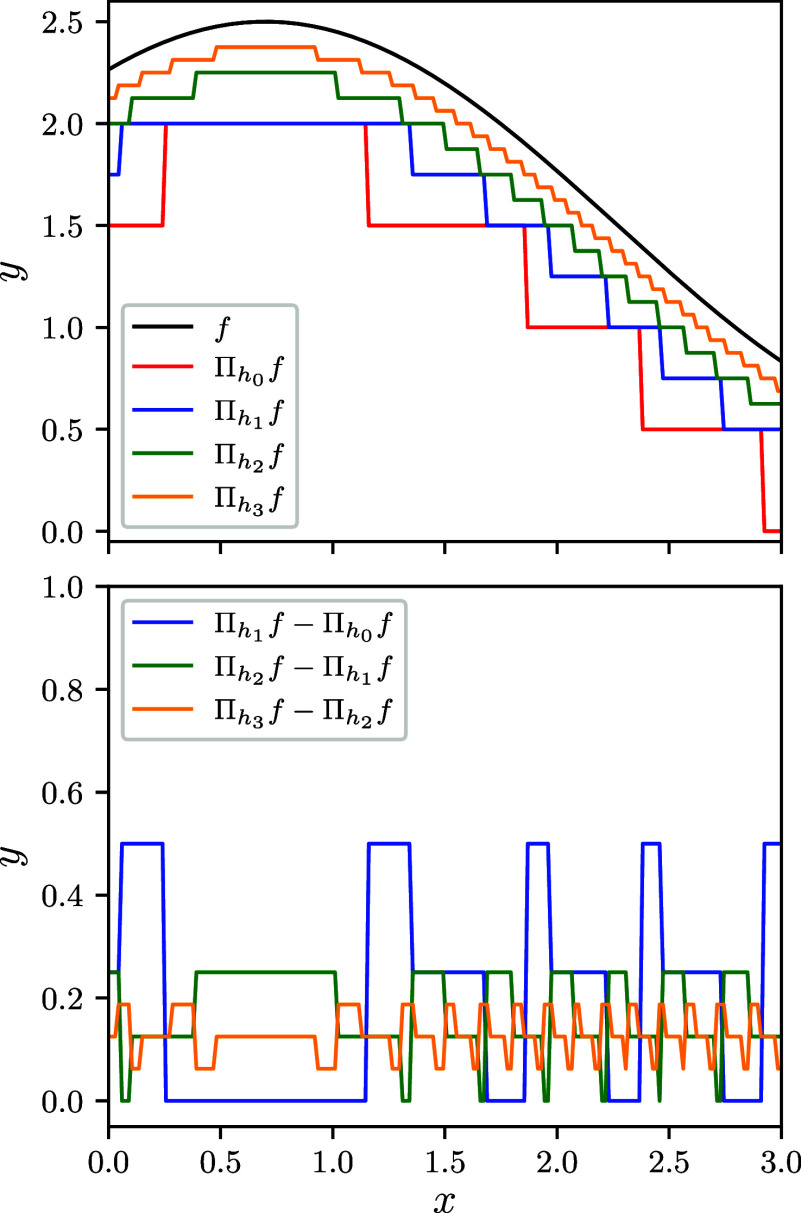
Illustration of multilevel Monte Carlo methods.

The *multifidelity Monte Carlo method* ^58^ generalizes the multilevel Monte Carlo method from a hierarchy of approximations to a single model to a hierarchy of different models which are correlated to a *high-fidelity model* of high accuracy and high computational cost. To this end, given a high-fidelity model *f*_hi_ and low-fidelity models *f*_1_, ..., *f*_*L*_, the Monte Carlo estimator given in eq 55 is based on sample numbers *N*_0_ ≤ ··· ≤ *N*_*L*_, i.i.d. samples $\xi_{1},...,\xi_{N_{l}}$ , and single-level estimators

57 $$E_{N_{0}}\left[f_{\mathrm{hi}}\right]=\frac{1}{N_{0}}\overset{N_{0}}{\underset{l=0}{\sum }}f_{\mathrm{hi}}\left(\xi_{l}\right)$$

58 $$E_{N_{l}}\left[f_{l}\right]=\frac{1}{N_{l}}\overset{N_{l}}{\underset{l=0}{\sum }}f_{l}\left(\xi_{l}\right)$$

59 $$E_{N_{l-1}}\left[f_{l}\right]=\frac{1}{N_{l-1}}\overset{N_{l-1}}{\underset{l=0}{\sum }}f_{l}\left(\xi_{l}\right)$$

and reads

60 $$E\left[f\right]\approx E_{N_{0}}\left[f_{\mathrm{hi}}\right]+\overset{L}{\underset{l=1}{\sum }}\alpha_{k}\left(E_{N_{l}}\left[f_{l}\right]-E_{N_{l-1}}\left[f_{l}\right]\right)≕E_{L}^{\mathrm{MF}}\left[f\right]$$

The sample numbers *N*_0_, ..., *N*_*L*_ and the *control variates* α_1_, ..., α_*L*_ ∈ R are determined from the Pearson correlation coefficients between the models, the variances of the models, and the computational cost of the various model evaluations (see ref 58).

### 3.5 Surrogate Models

Approximations to high-fidelity models which are cheap to evaluate are refereed to as *surrogate models*. These surrogate models play an important role in reducing the computational burden of sampling based methods, particularly when used as low-fidelity models in multifidelity methods.

#### 3.5.1 Surrogate Modeling and Machine Learning

From an abstract point of view, surrogate models are a mathematical model, the *surrogate method*, whose hyper-parameters are fitted to reference data, called the *training set*. Given suitable surrogate methods and training sets, surrogate methods often generalize well to other data. Clearly, stochastic collocation and polynomial chaos approaches (cf. section 3.4.4) as well as any machine learning method which infers models from training data can act as surrogate method. In the language of section 2.2, we may consider (ξ, *f*(ξ)) as the hypothetically exact training data in form of input–output pairs. In practice, we need to replace ξ by suitable approximations ξ_chem_, introducing a representation error, such that the surrogate method is a mapping ξ_chem_ → *f* ^comp^(ξ_chem_, η, *h*), where the model parameters η depend on the training data. Thus, the error of the surrogate method can be interpreted as a combination of a level of theory error and a numerics error. The error of surrogate methods, and particularly machine learning methods, themselves is an active field of research, with some methods providing exact mathematical results on the error behavior and other methods whose approximation properties are still not fully understood (see refs 106, 161, and 162 for comprehensive reviews).

#### 3.5.2 Gaussian Processes

A particularly popular instance of surrogate models with connections to Bayesian inverse problems are *Gaussian processes*,^163^ illustrated in Figure 9. A Gaussian process is a collection of random variables, where all finite subsets thereof have a joint Gaussian distribution. We write *f*(*x*, ω), where ω is a random event and *x* can be considered as an index to the random variables. It is well-known that a Gaussian process is fully specified by its mean m(x)=E[f](x) and its covariance function *k*(*x*, *x*′) = Cov­[*f*]­(*x*, *x*′) (cf. section 3.1.3 for their definition). Most importantly, the covariance function does not need to be a Gaussian in the sense of eq 13 and its multivariate analog. In fact, it is sufficient to satisfy symmetry, i.e., *k*(*x*, *x*′) = *k*(*x*′, *x*), and positive semidefiniteness, i.e., it holds

61 $$\overset{n}{\underset{i,j=1}{\sum }}k\left(x_{i},x_{j}\right)c_{i}c_{j}\geq 0$$

for all n∈N , *x*_1_, ..., *x*_*n*_, and *c*_1_, ..., *c*_*n*_. We write *f*(*x*, ω) ∼ $G\left(m\left(x\right),k\left(x,x^{\prime }\right)\right)$ or *f*(*x*, ω) ∼ $N\left(m\left(x\right),k\left(x,x^{\prime }\right)\right)$ and note that *f*(*x*, ω) has a natural representation in terms of a Karhunen–Loève expansion:

62 $$f\left(x,\omega \right)=m\left(x\right)+\overset{\infty }{\underset{l=1}{\sum }}\sqrt{\lambda_{l}}\psi_{l}\left(x\right)y_{l}$$

where $\left(\lambda_{l},\psi_{l}\right),l=1,2,...$ , are the eigenpairs of the integral operator

63 $$\left(Cg\right)\left(x\right)=\int k\left(x,x^{\prime }\right)g\left(x\right)\;dx^{\prime }$$

and

64 $$y_{l}\left(\omega \right)=\frac{1}{\sqrt{\lambda_{l}}}\int \left[f\left(x,\omega \right)-E\left[f\right]\left(x\right)\right]\psi_{l}\left(x\right)\;dx$$

are random variables. We note that the latter ones are usually not available in practice.

**Figure 9:**
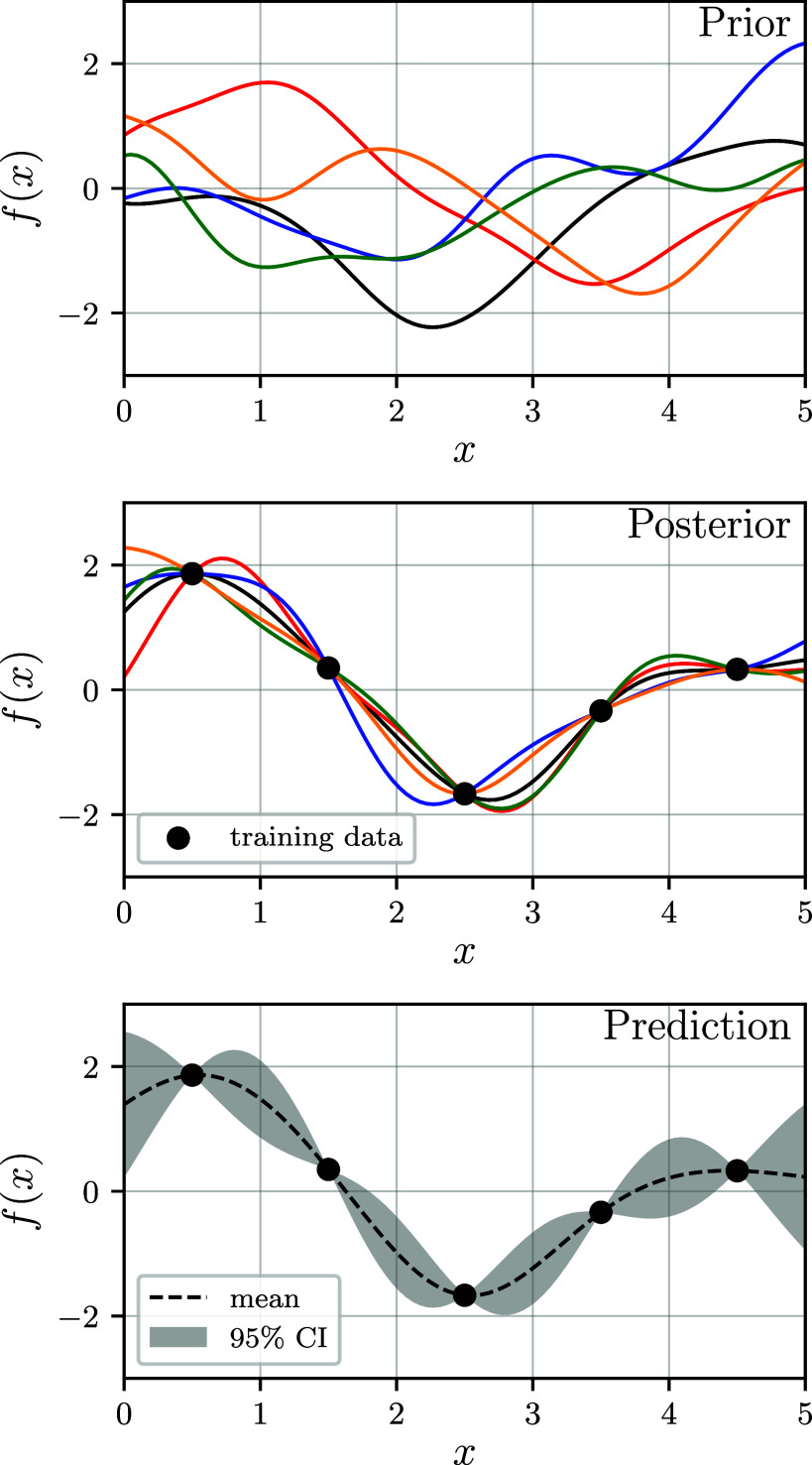
Visualization of Gaussian process regression, using a Gaussian kernel function with a mean of zero. The top panel shows samples drawn from the prior distribution. The central panel shows samples drawn from the posterior distribution with training points indicated by black dots. The bottom panel shows the predicted mean, with 95% confidence intervals indicated in gray.

Gaussian processes offer a natural capability to incorporate knowledge from measurements by using the Bayesian framework. Assuming as prior knowledge that our random variable *f*(*x*, ω) is a Gaussian process and that we have measurements

65 $$y_{i}=f\left(x_{i},\omega \right)+\varepsilon_{i}\left(\omega \right),,i=1,...,n$$

at certain locations *x*_1_, ..., *x*_*n*_ and subject to independently distributed Gaussian noise $\varepsilon_{i}∼N\left(0,\sigma^{2}\right)$ , the posterior distribution of *f* is explicitly given as a Gaussian process $G\left(m^{\delta }\left(x\right),k^{\delta }\left(x,x^{\prime }\right)\right)$ , with

66 $$m^{\delta }\left(x\right)=\mathrm{k̂}{\left(x\right)}^{T}{\left(\mathrm{K̂}+\sigma^{2}I\right)}^{-1}\left(y-m\right)$$

67 $$k^{\delta }\left(x,x^{\prime }\right)=k\left(x,x^{\prime }\right)-\mathrm{k̂}{\left(x\right)}^{T}{\left(\mathrm{K̂}+\sigma^{2}I\right)}^{-1}\mathrm{k̂}\left(x\right)$$

where *m* = [*m*(*x*_1_), ..., *m*(*x*_*n*_)]^T^ and

68 $$\mathrm{k̂}\left(x\right)=\left[\begin{array}{c} k\left(x_{1},x\right)\\ \vdots \\ k\left(x_{n},x\right) \end{array}\right]$$

69 $$\mathrm{K̂}=\left[\begin{array}{ccc} k\left(x_{1},x_{1}\right) & \cdots & k\left(x_{1},x_{n}\right)\\ \vdots & \ddots & \vdots \\ k\left(x_{n},x_{1}\right) & \cdots & k\left(x_{n},x_{n}\right) \end{array}\right]$$

This approach is also known as *Gaussian process prediction* or *interpolation*,^163^ *kriging*,^164,165^ or *kernel ridge regression*.^166,167^ See ref 168 for a thorough review in the context of chemistry.

### 3.6 Uncertainty Validation

Assessing the reliability of a prediction’s uncertainty is as important as assessing the reliability of the prediction itself. If a model’s estimated uncertainty is too small, one is at risk to overly trust the model’s predictions, resulting in running fewer (computer) experiments than necessary, thereby missing important examples. If a model’s estimated uncertainty is too large, on the other hand, one is at risk to overly distrust the model’s predictions, resulting in running more experiments than necessary, thereby wasting resources. Both situations are to be avoided by research facilities which have to act in the interest of both knowledge and economics, which stresses the importance of uncertainty validation.^169^

The uncertainty of a prediction usually refers to a confidence interval of a probability function for a given confidence level γ ∈ [0, 1]. The confidence level is also referred to as *nominal coverage probability* as it reflects the expected (nominal) relative frequency (probability) at which the true value lies within (is covered by) the associated confidence interval. The *actual* coverage probability refers to the observed (actual) relative frequency at which a benchmark lies within the associated confidence interval, which requires an out-of-sample/test set. Nominal and actual coverage probability match if, for an infinitely large test set,

70 $$\underset{M\to \infty }{\lim}M^{-1}\overset{M}{\underset{i=1}{\sum }}1\left(y_{i}\in I_{\gamma ,i}\right)=\gamma$$

The indicator function **1** (*y*_*i*_ ∈ *I*_*γ*__,*i*_) equals 1 if the benchmark value of the *i*th out-of-sample point, *y*_*i*_, lies within the confidence interval *I*_*γ*__,*i*_; if it lies outside the interval, the indicator function equals zero:

71 $$1\left(y_{i}\in I_{\gamma ,i}\right)=\{\begin{array}{ll} 1, & y_{i}\in I_{\gamma ,i}\\ 0, & y_{i}\notin I_{\gamma ,i} \end{array}$$

It is customary to report uncertainties (i.e., confidence intervals) for a given confidence level. In thermochemistry, for instance, γ = 0.95 is a convention.^170^ This corresponds to ca. 2 standard deviations if the underlying error distribution is normal. However, a single confidence level is insufficient in terms of uncertainty validation as the information that the actual coverage probability matches or does not match a given confidence level cannot be transferred to another confidence level. In the forecasting community, one speaks of a well-calibrated model if the actual coverage probability matches any confidence level,

72 $$\underset{M\to \infty }{\lim}M^{-1}\overset{M}{\underset{i=1}{\sum }}1\left(y_{i}\in I_{\gamma ,i}\right)=\gamma ,,\forall \gamma \in \left[0,1\right]$$

For instance, if a normal distribution of the true value with some mean value and variance is assumed, nominal and actual coverage probabilities will only match for any value of γ if the true underlying probability distribution is a normal distribution with the same mean value and the same variance.

Here, the term “uncertainty validation” refers specifically to the validation of uncertainty estimates themselves, and should not be confused with model validation as used in verification and validation (V&V) or VVUQ frameworks (cf. section 4.2).^171^

### 3.7 Dimension Reduction

As noted in the sections on quadrature methods (section 3.4.3) and spectral approaches (section 3.4.4), these approaches suffer from the curse of dimensionality. In other words, the number of required samples to obtain a desired accuracy grows exponentially with the number of parameters. This issue applies to surrogate models as reviewed in section 3.5 as well. Under certain circumstances, the curse of dimensionality can be mitigated using specialized methods, as for example discussed in sections 3.4.3 and 3.4.4. However, quite often it turns out that the behavior of a model is such that it can be (approximately) described by significantly less parameters than given. When a model is (approximately) reparametrized to depend on fewer parameters, this is referred to as *dimension reduction*. Significant computational gains can be made when the dimensionality of a model is reduced before applying any of the above-mentioned methods.

Dimension reduction is an active research area, particularly in machine learning, whose review is beyond the scope of this review. We content ourselves by mentioning two of the most popular approaches are based on principal component analysis and global sensitivity analysis. Both approaches provide us with a linear decomposition where parameters with small contribution can be identified and discarded. For further details on dimension reduction approaches, we refer the reader to refs 172–175 .

### 3.8 Software for Uncertainty Quantification

Along with the emergence of UQ in the research fields outlined above and beyond, various software packages have been developed in the context of UQ. Available software presently offers a broad range of tools, covering simple basic statistical analyses as well as advanced approaches to specific applications. Below, we provide a nonexhaustive list of UQ software and libraries that are potentially applicable in the field of *in silico* chemistry. The tools are listed alphabetically, including a short summary highlighting their main features.

- **ChaosPy**:^176^ Open-source python library providing specific tools for polynomial chaos expansions and advanced MC sampling. A focus is put on its compatibility with other (python) libraries for UQ.
- **DAKOTA**:^177^ Large open-source C++ project with broad functionality between optimization (both gradient and nongradient methods), UQ (sampling and reliability as well as stochastic expansion methods), parameter analysis using nonlinear least-squares optimization, and sensitivity analysis (design of experiments and parameter study methods). One of its main advantages is the possible combination of the available methods in a user-defined fashion.
- **EasyVVUQ**:^171,178^ Open-source python library focusing on UQ for MD simulations, providing, for example, polynomial chaos expansions, stochastic collocation, and sensitivity analysis functionality. Part of the SEAVEA toolkit.
- **MUQ** ^179^ Open-source python/C++ library for inverse and forward UQ, featuring various MCMC-based sampling techniques, regression and prior modeling via Gaussian processes, as well as polynomial chaos expansions and nonlinear optimization.
- **NIST Uncertainty Machine**:^180^ Open-source web-based application that can be operated to assess the measurement uncertainty of an output quantity by means of Gaussian error propagation and MC sampling. The output quantity is required to be a real, explicitly known function of any number of input quantities which are modeled as random variables.
- **OpenTURNS**:^181^ Open-source python/C++ library for multivariate probabilistic data modeling, including Bayesian calibration and sensitivity analysis.
- **physical_validation**:^182^ Open-source python library that offers a wide range of tests that can be applied to MD and MC simulations *a posterior* to validate their physical correctness (e.g, in terms of chosen cutoffs, thermostat, simulation time step etc.) Moreover, functionality for checking code-correctness of MD software is offered, initially developed for GROMACS^183^ but designed to be extended to other software.
- **PyApprox**:^184^ Open-source python/C++ library for high-dimensional approximation and UQ. Special features include tools for building surrogate models, sparse grid interpolation, multifidelity approximations, Bayesian inference methods, and more.
- **SALib**:^185^ Open-source python library for global sensitivity analysis of general computational models.
- **SEAVEA toolkit**: Open-source collection of tools for VVUQ with applications on large-scale computing infrastructures (www.seaveatk.org). Includes, for example, EasyVVUQ and is a successor of the VECMA toolkit.^186^
- **SMCPy**:^187^ Open-source python library developed to enable UQ via parallel, sequential MC sampling. As its key features, it offers an alternative to the MCMC method when treating Bayesian inference problems and an unbiased marginal likelihood estimation in terms of Bayesian model selection.
- **Sparse Grids Matlab Kit** ^188^ Matlab implementation of sparse grids,^54^ designed to approximate functions of high order. This tool is applicable for surrogate model-based UQ. Review and comparison to other software packages.
- **UncertainSCI**:^189^ Open-source python tool for UQ in the field of biomedical simulations, focusing on the propagation of input uncertainties on model outputs by polynomial chaos expansions.
- **UM-Bridge**:^190^ Open-source python/C++ toolkit designed for combining any kinds of computational models with various statistical/optimization methods of UQ. This is achieved by containerization of software, enabling the coupling of models and UQ methods from different programming languages and computational environments, with the intention to provide a unified interface for UQ methods from all fields of science.
- **UQLab**:^191^ Open-source Matlab framework for UQ in applied science and engineering, providing software that can be combined in a user-defined and modular fashion. Supported UQ techniques include among others: Bayesian model calibration, sensitivity analysis, support vector machines, polynomial chaos expansions.
- **UQTk**:^192^ Open-source python/C++ toolkit comprising several libraries for UQ on numerical models, focusing on polynomial chaos expansions, uncertainty propagation, sensitivity analysis and Bayesian inference.
- **Uranie**:^193^ Open-source C++ software with python support, designed to process and handle large data when performing UQ. Specifically, it supports design of experiments and surrogate model generation, sensitivity analysis, calibration and parametric optimization as well as dedicated visualization tools.
- **WESTPA**: Weighted Ensemble Simulation Tool^194,195^ and tutorial for rare event sampling.^196^

## 4 Applications of Uncertainty Quantification in *In Silico* Chemistry

### 4.1 Energy Uncertainties

#### 4.1.1 Wavefunction-Based Quantum Chemistry

Wavefunction-based quantum chemistry provides methods for approximately solving the nonrelativistic many-electron Schrödinger equation within the Born–Oppenheimer approximation (i.e., for a fixed molecular structure with nuclear coordinates ***R***_1_, ***R***_2_, ...):

73 $${Ĥ}^{\mathrm{el}}\Psi_{n}\left(x_{1},...,x_{N}\right)=E_{n}^{\mathrm{el}}\Psi_{n}\left(x_{1},...,x_{N}\right)$$

with the electronic Hamiltonian *Ĥ* ^el^, the electronic wavefunction Ψ_*n*_, and the combined spatial and spin coordinates ***x***_*i*_ = (***r***_*i*_, *s*_*i*_) of the electrons. Here, we will mainly consider the ground-state electronic energy *E*_0_^el^(**R**_1_, **R**_2_, ...) as the quantity of interest.

Single-reference wavefunction-based quantum-chemical methods, such as configuration interaction (CI) and coupled-cluster (CC) methods, generally use the Hartree–Fock approximation as their starting point, in which the electronic wavefunction is approximated by a single Slater determinant Ψ_SD_, which is formed from the occupied Hartree–Fock orbitals ϕ_*i*_(***r***). The Hartree–Fock orbitals are in turn approximated by expanding them in suitable atom-centered basis functions χ_μ_(***r***):

74 $$\phi_{i}\left(r\right)=\overset{N_{\mathrm{bas}}}{\underset{\mu =1}{\sum }}c_{\mu }^{\left(i\right)}\chi_{\mu }\left(r\right)$$

Besides the occupied orbitals, the solution of the Hartree–Fock equations also provides a set of unoccupied (virtual) orbitals, which can be used to construct additional Slater determinants Φ_*ij*···_^*ab*···^ by replacing the occupied orbitals *i*, *j*, ... in the Hartree–Fock determinant by virtual orbitals *a*, *b*, .... The many-electron wavefunction is then expanded in a suitable basis of these Slater determinants as

75 $$\Psi^{\mathrm{el}}\left(x_{1},...,x_{N}\right)=\underset{n}{\sum }C_{n}\Phi_{n}\left(x_{1},...,x_{N}\right)$$

where the expansion is generally terminated based on the excitation level (i.e., the maximum number of occupied orbitals that are replaced by virtual orbitals). Multireference methods^197–200^ perform the optimization of the molecular orbitals simultaneously with the determination of the expansion coefficients of the Slater determinants, and usually define their many-electron wavefunction in terms of an active space of orbitals.

For further details on specific quantum-chemical methods, we refer the reader to ref 95. We note that explicitly correlated methods,^201^ which extend the ansatz of eq 75 by including terms that depend on the interelectronic distance | ***r***_*i*_ – ***r***_*j*_|, are nowadays widely used. The following discussion also applies to such explicitly correlated methods.

When considering errors in wavefunction-based quantum chemistry, one usually considers the exact nonrelativistic ground-state energy of eq 73 as the reference. Therefore, uncertainties in the calculated ground-state energy will be solely due to the approximation introduced by the quantum-chemical methods. Generally, there are two main sources of errors in approximate wavefunction-based quantum-chemical methods: First, the incompleteness of the basis set that is used for expanding the one-electron orbitals (see eq 74). Second, the incompleteness of the basis of Slater determinants that are used for expanding the many-electron wavefunction (see eq 75). In principle, both of these approximations can be controlled and systematically improved (see Figure 10). The exact ground-state energy of the nonrelativistic Schrödinger equation can be obtained with a full-CI calculation in an infinitely large one-electron basis set. Here, it is important to note that neither full-CI calculations in a finite one-electron basis set or calculations using a truncated expansion in Slater determinants in a complete one-electron basis set provide the exact solution, but both limits have to be approached simultaneously.

**Figure 10:**
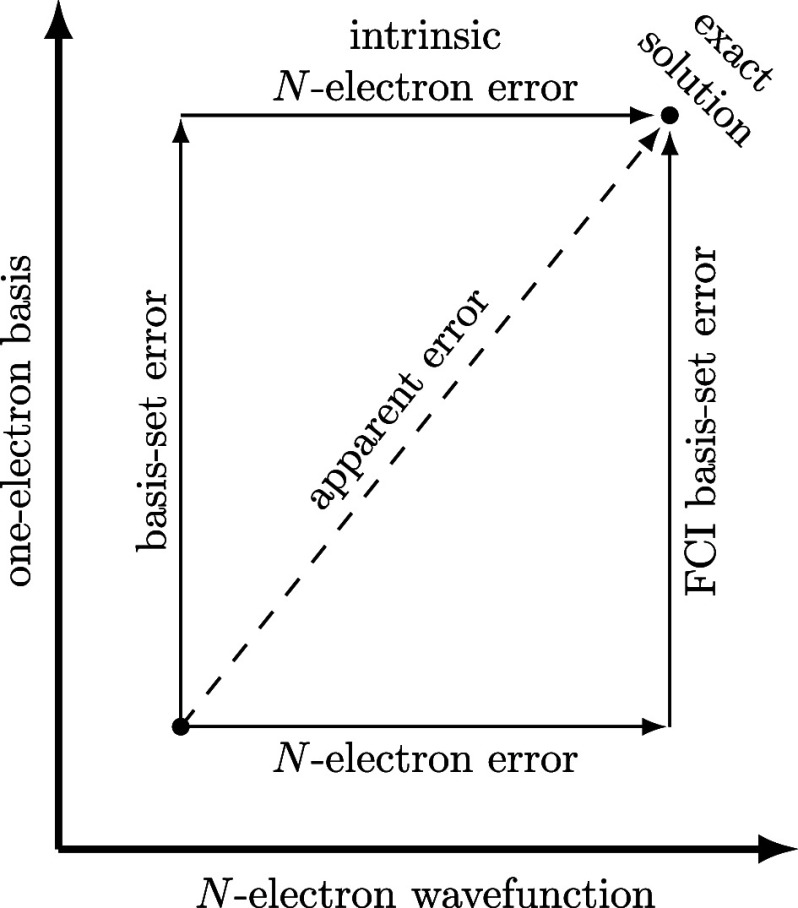
Errors in quantum-chemical calculations. Adapted from ref 95.

However, this exact solution (or an energy that is within the required numerical accuracy) is hardly achievable in practice for molecules, except for the smallest systems (see, e.g., refs 202–204 ). Nevertheless, numerous benchmarking studies have demonstrated the systematic convergence of total energies as well as atomization energies and reaction energies toward experimental reference values. For a particularly systematic example of such a study, we refer the reader to Chapter 15 of ref 95.

##### Error Estimation by Extrapolation

For quantum-chemical calculations using accurate wavefunction-based methods, one can try to systematically approach the exact solution in calculations for individual systems. One common approach to this end is based on the use of extrapolation schemes, in which one performs calculations with increasing basis set sizes and uses these to extrapolate to the basis set limit. This can be further combined with an extrapolation of the correlation contributions by performing calculations that systematically increase the number of included Slater determinants (e.g., by increasing the excitation level in a CI expansion or a CC ansatz). For a review of methods for highly accurate quantum-chemical calculations, including a comprehensive overview of extrapolation schemes and composite methods, we refer the reader to ref 205.

Such extrapolation schemes also provide a basis for estimating the remaining error of the extrapolated energies. For instance, one can use the difference between the extrapolated energies and those of the calculations with the largest basis set and correlation level. Alternatively, the error can be estimated by comparing the results of different extrapolations. The details will depend on the specific composite and/or extrapolation scheme.

An example of such an approach can be found in ref 206, where the basis-set error of CCSD­(T) calculations is estimated by comparing the predictions of a basis-set extrapolation scheme to those obtained with the largest basis set. A general scheme using random walks, that can be used to estimate the uncertainty of energies obtained by extrapolating to the complete basis-set limit, has recently been put forward by Lang et al.^207^

To also include uncertainties arising from electron correlation, Schuurman et al.^208^ performed a focal-point analysis to obtain accurate estimates of the heats of formation for NCO, HNCO, HOCN, HCNO, and HONC. They used the convergence behavior and internal consistency of their results to estimate that the uncertainty in their predictions amounts to less than 0.2 kcal mol^–1^. Also using a focal point analysis, Hajgató et al. calculated the singlet–triplet gaps in polyacenes, and estimated the uncertainty in their predictions by comparing different extrapolations.^209^ An elegant and detailed protocol for estimating uncertainties within a composite scheme has been put forward by Bakowies,^210,211^ who developed a sophisticated model for the different relevant sources of errors.

A very instructive example showcasing systematic error estimation for highly accurate quantum-chemical calculations was recently presented by Jeziorski and co-workers, who calculated the three-body potential of neon.^212^ They carefully estimated the error in each term by considering the difference between the two largest employed basis sets. Another example are highly accurate calculations of the potential energy curve of the chromium dimer by Larsson et al.^213^ They combine an estimate of the correlation error with a small basis set with the estimated uncertainty of their basis set extrapolation.

While these examples show that for wavefunction-based quantum chemistry, the possibility to systematically approach the exact solution can be used to estimate uncertainties, we are not aware of any recommendations or accepted protocols for reporting estimated errors in such calculations.

##### Multireference Diagnostics

We note that the approaches discussed above are mostly restricted to single-reference methods. However, such approaches fail in cases that are dominated by static correlation (i.e., in which the dominating contribution to the exact electronic wavefunction is not from a single determinant, but from several determinants with similar weights). In such cases (e.g., for many open-shell transition metals), multireference methods^197–200^ are required.

While for single-reference methods, a clear hierarchy can be defined based on the excitation level, this is a nontrivial problem when selecting the active space in multireference methods (for attempts to overcome this limitation, see refs 214–217 ), which often precludes the application of systematic extrapolation schemes for error estimation.

There have been many attempts to devise diagnostic measures that allow one to detect whether single-reference methods are applicable for a certain system (for an overview, see refs 218 and 219). Within the Weizmann W4 composite method,^220,221^ the percentage of the total atomization energy (TAE) due to quadruple and quintuple excitations,

76 $$\%\mathrm{TAE}\left[{\mathrm{T̂}}_{4}+{\mathrm{T̂}}_{5}\right]=\frac{\mathrm{TAE}\left[\mathrm{CCSDTQ}5\right]-\mathrm{TAE}\left[\mathrm{CCSDT}\right]}{\mathrm{TAE}\left[\mathrm{CCSDTQ}5\right]}\times 100\%$$

or the %TAE due to perturbative triples excitations,

77 $$\%\mathrm{TAE}\left[\left(T\right)\right]=\frac{\mathrm{TAE}\left[\mathrm{CCSD}\left(T\right)\right]-\mathrm{TAE}\left[\mathrm{CCSD}\right]}{\mathrm{TAE}\left[\mathrm{CCSD}\left(T\right)\right]}\times 100\%$$

where TAE­[X] is the TAE calculated with method X, was used as an indicator of multireference character and considered as an estimate of the uncertainty in the calculated total atomization energies. This is in the same spirit as similar uncertainty estimators for extrapolation schemes and composite methods discussed above. In ref 221, it was observed that %TAE[*T̂*_4_+*T̂*_5_] and %TAE[(T)] show a very good correlation, and consequently, it has been suggested to use %TAE[(T)] as a diagnostic of multireference character and as an *a priori* estimate for the contribution of higher excitation levels.

Many different measures have been put forward as multireference diagnostics.^222^ For coupled-cluster methods, the use of the norm of the singles amplitudes, *T*_1_,^223^ is widespread, and related diagnostics based on the coupled-cluster amplitudes have also been put forward.^224–227^ For coupled-cluster methods, it was recently proposed that the extent of nonhermiticity of the one-electron density matrix can be used to estimate the error in the correlation energy.^228^ Another class of multireference diagnostics is based on occupation numbers from finite-temperature DFT calculations or on natural occupation numbers from MP2 or CASSCF calculations.^229–234^ Such indicators, which are usually cheeper to calculate than %TAE[(T)], have been discussed as predictors of the latter (i.e., as quantitative uncertainty estimate),^235^ or at least as a qualitative indicator whether single-reference methods are applicable.

However, the correlation between different multireference diagnostics is generally rather poor.^219,235,236^ Machine learning approaches, which combine different easily computable multireference diagnostics as well as geometric descriptors, have been shown to be promising in predicting multireference character as measured by %TAE[(T)] for transition metal complexes.^237–239^ This has been applied to exclude problematic (i.e., high uncertainty) cases in virtual high-throughput screening.^239^

##### Accuracy Predictor for MP2

For Møller–Plesset second-order perturbation (MP2) theory applied to noncovalently bonded complexes, Vuckovic et al. have developed an accuracy predictor^240^ by employing the adiabatic connection. The adiabatic connection considers the total energy as a function of a scaled electron–electron interaction, while keeping the electron density constant. The authors point out that MP2 theory approximates this adiabatic connection curve by a straight line, and propose that the deviation of the exact adiabatic connection curve from linear behavior can be used to estimate the accuracy of MP2 interaction energies. They show that for weakly interacting complexes, the exact adiabatic connection curve can be approximated by interpolating between the weakly and strongly interaction limits^241^ and by using an approximation for the latter.^242^ The resulting predictor can be calculated with no additional effort and correlates well with the errors in the MP2 interaction energies in comparison to high-level reference data.

##### Rigorous Error Bounds

Since the electronic Schrödinger equation is a (Hermitian) eigenvalue problem, it is possible to apply error bounds on approximate eigenvalues to quantum-chemical methods for its approximate solution. In particular, the Kato–Temple theorem (see, e.g., ref 243) states that for an approximate normalized eigenvector Ψ̃ and the corresponding approximate eigenvalue *Ẽ* = ⟨Ψ̃|*H̃*|Ψ̃⟩, the distance to the closest exact eigenvalue *E* is bounded by

78 $$\left|Ẽ-E\right|\leq \frac{∥r{∥}^{2}}{\delta }$$

where δ is the distance from *E* to the remaining eigenvalues (i.e., |*E* – *E*_*j*_| ≥ δ for all other eigenvalues *E*_*j*_) and *r* is the residual, given by

79 r=ĤΨ̃−ẼΨ̃

Such error bounds have been applied for the analysis of CI^244^ as well as CC methods.^245^ For a detailed discussion, we refer the reader to ref 246 and other works mentioned therein. While the formal analysis of the errors is valuable by itself, such rigorous error bound for energies from wavefunction based quantum-chemical methods have to the best of our knowledge not been used to calculate uncertainties for individual calculations, which we believe is for two reasons. First, the calculation of the norm of the residual, ∥*r*∥^2^, is difficult in practice, as it has to be represented outside of the (finite) Hilbert space that is used in the calculations. In particular for the many-electron basis, this presents a insurmountable obstacle. Second, error bounds for the total energy are rarely relevant in practice, as chemistry is concerned with relative energies. In computational chemistry, the calculation of energy differences always profits from systematic error cancelation, even with the most accurate methods.^205^ Therefore, uncertainties in relative energies that can be derived from error bounds for total energies are rarely useful.

#### 4.1.2 Density Functional Theory

Kohn–Sham density functional theory (KS-DFT) is the workhorse of quantum chemistry, mostly because of its favorable cost–accuracy ratio.^247–251^ It approximates the total electronic energy as a functional of the KS orbitals {*ϕ*_*i*_(***r***)}, yielding the electron density $\rho \left(r\right)=\underset{\mathrm{occ}}{\sum }|\phi_{i}\left(r\right){|}^{2}$ :

80 $$E\left[\left\{\phi_{i}\right\}\right]=T_{s}\left[\left\{\phi_{i}\right\}\right]+V_{\mathrm{nuc}}\left[\rho \right]+J\left[\rho \right]+E_{\mathrm{xc}}\left[\rho \right]+E_{\mathrm{NN}}$$

where *T*_s_[{*ϕ*_*i*_}] is the noninteracting kinetic energy, *V*_nuc_[ρ] is the electron–nuclear attraction energy, *J*[ρ] is the classical Coulomb repulsion of the electrons, *E*_NN_ is the nuclear repulsion energy, and *E*_xc_[ρ] is the exchange–correlation (xc) energy. In practice, the latter is treated using approximate functionals of the electron density (and possibly additional ingredients, such as occupied and/or virtual Kohn–Sham orbitals). Minimization of the above energy functional leads to the one-electron KS equations, from which the KS orbitals are determined.

When considering the exact solution of the nonrelativistic Schrödinger equation for a fixed molecular structure as benchmark, uncertainties in the energies calculated with DFT^66^ arise from the approximate xc functional, the incompleteness of the basis set used for the expansion of the KS orbitals as well as, for instance, numerical integration, convergence thresholds, the use of pseudopotentials to represent core electrons, and Brillouin zone sampling in the case of periodic boundary conditions. The classification of these error sources as level of theory uncertainties and as numerics uncertainties is not always clear and varies in different contexts (see section 2.2)

##### Rigorous Bounds for Numerical Errors in DFT

While in principle numerical errors in DFT calculations are well-controllable by systematically investigating the convergence with respect to the relevant parameters, such as the size of the basis set or convergence thresholds, the choice of these parameters is not trivial in practice. A large-scale comparison of periodic boundary condition DFT calculations for solids revealed striking differences between the implementations in different DFT codes (which each come with different numerical approximations and default choices).^252^ Even though these differences could be reconciled, this highlights the importance of systematically estimating and controlling numerical errors in DFT calculations^253^

A comprehensive discussion of the sources of numerical errors in plane-wave DFT calculations can found in ref 99. Herbst et al. implemented rigorous error bound for plane-wave DFT calculations, excluding contributions of the SCF iterations, i.e., for the nonself-consistent Kohn–Sham equations. To this end, the authors applied the Kato–Temple bound (eq 78). The calculation of the residual is simplified when using a plane-wave basis set, and an upper bound can be calculated using a larger plane-wave cutoff. This is combined with an lower bound for the energy gap between eigenvalues and with a bound on the numerical error, which is tracked by using interval arithmetics in their implementation. The resulting error bars present a rigorous bound on the error in the orbital energies within the considered model.

Further mathematical analyses of convergence and error bounds are available for DFT calculations,^254,255^ including error bounds for the basis set error with plane-waves,^256^ in augmented plane-wave approaches,^257^ and for atomic orbital-like basis functions.^258^ Also for error sources that are not treated in the implementation of Herbst et al.,^99^ such as the SCF procedure^259,260^ and *k*-point sampling,^261^ the mathematical tools for establishing rigorous error bounds for total energies are available. However, as for wavefunction methods, it is unclear whether these translate to useful error bounds on relative energies.

##### Error of Exchange–Correlation Approximations

A main source of error in DFT calculations is the approximate exchange–correlation (xc) functional *E*_xc_[ρ]. A plethora of approximations are available,^262–264^ and while numerous studies exist that benchmark their accuracy for carefully devised test sets (see, e.g., refs 265 and 266), it is generally unclear how accurate a chosen approximate xc functional will be for a specific molecule. Here, we will focus on approaches that are able to assign an uncertainty to the energy calculated *for a specific molecule*.

One simple measure that often gives an indication of the accuracy of a DFT calculation is the variation with the percentage of exact exchange λ that is included in the xc functional. This can be captured by measures such as the *B*_1_ diagnostic:^267^

81 $$B_{1}=\frac{\mathrm{TAE}\left[\mathrm{BLYP}\right]-\mathrm{TAE}\left[B1\mathrm{LYP}\right]}{n_{\mathrm{bonds}}}$$

where TAE­[BLYP] is the total atomization energy calculated with the nonhybrid functional BLYP, TAE­[B1LYP] is the total atomization energy calculated with the hybrid functional B1LYP, which contains 25% of exact exchange, and *n*_bonds_ is the number of covalent bonds in the molecule. The *A*_*λ*_ diagnostic^230^ uses a different normalization. When, for instance, applied to the PBE0 functional (with λ = 0.25), it is defined as

82 $$A_{25}\left[\mathrm{PBE}\right]=\frac{1.00}{0.25}\frac{\mathrm{TAE}\left[\mathrm{PBE}\right]-\mathrm{TAE}\left[\mathrm{PBE}0\right]}{\mathrm{TAE}\left[\mathrm{PBE}\right]}$$

While such measures can help to identify molecules for which a large uncertainty can be expected for DFT calculations in general, they do not provide an accuracy that is related to a specific choice of xc approximation. Therefore, they cannot be used to guide the selection of a specific xc approximation, or help identify a suitable amount of exact exchange.

Many approximations to the xc functional contain empirical parameters ***a*** that are fitted such that the root-mean-square error (or a similar cost function *C*(***a***)) for a training set is minimized. The framework for Bayesian error estimation functionals (BEEFs) in DFT originally proposed by Mortensen et al.^268^ extends the best-fit set of parameters ***a***_0_ by a probability distribution *p*_***a***_ for the empirical parameters in the xc approximation, i.e., instead of a single (best-fit) approximate functional, one uses an ensemble of functionals. The BEEF framework assumes that for parameters that enter the xc functional linearly,

83 $$p_{a}\propto \exp\left(-C\left(a\right)/T\right)$$

where the spread of the distribution is determined by the ensemble temperature *T* = 2*C*(***a***_0_). For details on the determination of this probability distribution, we refer the reader to refs and 63 and^268^. By sampling from this probability distribution and performing DFT calculations with different sets of parameters, it becomes possible to obtain a probability distribution for the resulting energies, from which a mean and a variance can be assigned to each result. An extension of this theoretical framework was put forward by Aldegunde et al.,^269^ who apply a relevance vector machine learning model and obtain probability distributions for the model parameters using Bayesian inference (see section 3.2.2).

Within the BEEF framework, Wellendorff et al. developed the BEEF-vdW GGA functional^270^ as well as the mBEEF meta-GGA functional.^271^ Both restrict the parameter space to linear parameters in the GGA enhancement factor of the exchange functional. The sampling of the parameter space is only performed post-SCF, i.e., the electron density is not reoptimized for each set of parameters, which allows for an efficient evaluation of the uncertainty. By sampling the parameter distribution, the BEEF framework makes it possible to not only obtain uncertainties for individual energies, but also for energy differences and other properties derived from the energy. This has been exploited to assess the uncertainties in harmonic vibrational frequencies^272^ (see section 4.3.3), finite-temperature thermodynamic properties of solids,^273^ as well as for various applications of DFT in heterogeneous catalysis.^274–281^

While these BEEF functionals provide a possibility for estimating uncertainties due to the xc approximation, it is unclear whether the parametric uncertainty in the BEEF model is able to correctly predict the error distribution of these functional.^67,282,283^ Therefore, Reiher and co-workers developed a system-specific long-range corrected PBE functional within the BEEF framework.^63,284^ They reoptimize five (linear and nonlinear) parameters in this functional for a specific class of systems of interest, but only use the one linear parameter for estimating the uncertainty. They stress that a system-specific parametrization is crucial not only for improving the accuracy of the xc functional for a problem at hand, but also for the reliable estimation of uncertainties.

Walker et al.^285^ used a latent variable model to predict uncertainties in DFT-calculated energies. For the energies of the species of interest *y*, they performed DFT calculations using four different approximate xc functionals and fitted a model of the form

84 y=Wz+μ+e

where ***z*** is a normally distributed latent variable, **μ** are the mean values predicted over the considered functionals, and ***e*** is a zero-mean Gaussian distributed noise with specific variances **Ψ**. The unknown parameters ***W*** and **Ψ** are obtained by maximizing a log-likelihood function such that the model is consistent with the considered ensemble of approximate xc functionals. The uncertainties provided by this model can then be propagated to assign uncertainties to other quantities of interest, such as turnover frequencies, apparent activation barriers, and reaction orders. This was applied to model the various experimental kinetic data^285^ and to identify active sites^286^ of the water–gas shift reaction over Pt-based catalysts. A similar approach has been applied to investigate ethane dehydrogenation and hydrogenolysis on Pt surfaces.^287^

An alternative approach to quantifying the uncertainties due to the xc approximation is the parametrization of Δ-machine learning^288^ models that predict the difference between approximate DFT calculations and accurate high-level quantum-chemical calculations. The simplest case is a linear regression model,

85 X=βT+ϵ

which predicts the experimental values *X* from the calculated values *T*, and where β is a linear regression coefficient and ϵ is a random error with zero mean. Lejaeghere et al.^289^ applied such a model to the prediction of cohesive energies, bulk moduli, and elastic constants of elemental crystals, and assign uncertainties to the predictions of solid-state DFT calculations with approximate xc functionals. Similarly, Pernot et al.^290^ employed linear calibration models to access the prediction uncertainty of properties from solid-state DFT calculations. Furthermore, uncertainties have been assigned^291–294^ to linear correction models that provide corrections to DFT energies (e.g., by interpolating between DFT and DFT-U)^295,296^ in solid-state chemistry.

Simm and Reiher^297^ applied Gaussian process regression to learn the difference between PBE and extrapolated MP2 atomization energies, and apply this for the exploration of chemical reaction networks (see also section 4.3.1). To this end, their model uses geometry-dependent descriptors. While the Δ-machine learning model provides a prediction of the error in the DFT calculation, they actually use the energies corrected by this model (i.e., approximations to the high-level MP2 results) as their quantity of interest, and employ the uncertainty provided by the Gaussian process (see section 3.5.2) to assess the accuracy of their model. If this uncertainty becomes too large, additional high-level calculations are performed to improve upon the model in a rolling reparametrization (active learning).

For an extensive test set of binary and ternary oxides, Yuk et al.^298^ trained a random forest regression model to predict the error with respect to experimental reference data for four approximate xc functionals. As features, their model uses descriptors from materials informatics, specifically the fractional ionicity, charge per valence electron, valence electrons per atomic number, Pauling electrostatic strength of the metal–oxygen bond, mass density, oxygen fractional occupation, and element specific DFT ionization errors. Similarly, but without reference to experimental or computational reference data, Alfonso-Ramos et al. trained a machine learning model using structural fingerprints as features to predict the variance in the activation energies of pericyclic reactions calculated by 20 approximate xc functionals.^299^

For transition metal complexes, Kulik and co-workers^300^ developed a Δ-machine learning model that predicts the difference between the spin-state energies calculated with DFT using an approximate xc functional and with DLPNO-CCSD­(T), i.e., the error due to the xc functional. Their model relies on descriptors that depend on the electron densities calculated for the high-spin and low-spin state with a specific xc functional (B3LYP). By parametrizing such models for 48 different approximate xc functionals, they are able to recommend the best-performing xc functional for a specific transition metal complex.

##### Errors of Dispersion Corrections

Since semilocal xc approximations are known to miss dispersion interactions, such xc functionals are mostly combined with semiclassical dispersion corrections.^301,302^ These corrections model the error in the dispersion energy via an atomic-pairwise potential in terms of *C*_6_ (and possibly *C*_8_, *C*_10_, ...) coefficients, in combination with an empirical damping function in the short and medium range. This damping function contains empirical parameters that are fitted to experimental or high-level computational reference data.

Semiclassical dispersion interactions provide a well-defined model that can be used as a test case for uncertainty quantification in computational chemistry. For first-generation (DFT-D) dispersion interactions,^303,304^ Hanke^305^ assessed the sensitivity of the dispersion energy with respect to their different parameters. Using *ad hoc* estimates of uncertainties in these parameters, they investigated the resulting uncertainties in calculated binding energies. Conversely, by comparing to reference data, they estimated the uncertainty in a global parameter of the dispersion correction.

For the D3 dispersion correction,^306^ Weymuth et al.^307^ performed an in-depth statistical analysis of the sensitivity with respect to its empirical parameters. They performed a bootstrapping analysis (section 3.4.2) to assign uncertainties both to these parameters and to the predicted dispersion energies. Moreover, they performed a jackknife analysis to assess the quality of the reference dataset that was used to parametrize D3.

Proppe et al.^308^ trained a Gaussian process model for the dispersion energy using the interaction energy difference between DFT and coupled-cluster calculations as training data, using only structural information that is also available to the D3 model as input features. This Gaussian process provides not only a prediction of the dispersion energy, but also for the associated uncertainty. This active-learning strategy^309,310^ can, in turn, be used to refine the model in regions where this uncertainty becomes too large.

#### 4.1.3 Semiempirical Quantum Mechanics

Semiempirical quantum-chemical (SQM) methods^311–313^ employ a Hamiltonian akin to the one appearing in the HF or DFT formalism, usually in a minimal basis set, and approximate the parameters appearing in it using simplified models. These parameters are generally obtained by a fit to suitable reference data, which makes then amenable to uncertainty quantification.

One attempt at quantifying uncertainties in the PM7 method was undertaken by Oreluk et al.^314^ Using the Bound-to-Bound Data Collaboration framework,^315–317^ they assigned feasible intervals to each parameter that are consistent with the training data, and propagated these to error intervals for the heats of formation of linear alkanes. They found that the accuracy of the training data was preserved for the predicted heats of formation within bounds of chemical accuracy if predictions were made for the molecules of comparable size, but that the error grows linearly with the relative size of the molecules.

Finally, we mention that Δ-machine learning models based on Gaussian processes as discussed in section 4.1.2 for DFT are also applicable in combination with SQM methods.^297^

#### 4.1.4 Classical Force Fields

Classical force fields model the ground-state potential energy surface using classical interaction terms. In their most common form,^96,97,318^

86 $$E_{\mathrm{FF}}\left(R_{1},...,R_{N}\right)=\frac{1}{2}\overset{\mathrm{bonds}}{\underset{i}{\sum }}k_{i}{\left(r_{i}-r_{i}^{0}\right)}^{2}+\frac{1}{2}\overset{\mathrm{angles}}{\underset{j}{\sum }}k_{j}^{\theta }{\left(\theta_{j}-\theta_{j}^{0}\right)}^{2}+\overset{\mathrm{torsions}}{\underset{n}{\sum }}\cos\left(n_{n}\omega_{n}-\gamma_{n}\right)+\underset{I}{\sum }\underset{J>I}{\sum }\frac{q_{I}q_{J}}{r_{IJ}}+\underset{I}{\sum }\underset{J>I}{\sum }4{\text{ϵ}}_{IJ}\left[{\left(\frac{\sigma_{IJ}}{r_{IJ}}\right)}^{12}-{\left(\frac{\sigma_{IJ}}{r_{IJ}}\right)}^{6}\right]$$

they include bonding terms, which depend on bond lengths *r*_*i*_, bond angles θ_*j*_, and torsional angles ω_*n*_, as well as nonbonding electrostatic and van der Waals interactions, which depend on interatomic distances *r*_*IJ*_ = | ***R***_*I*_ – ***R***_*J*_|. Such force fields form the basis of molecular dynamics simulations that can be used to determine finite-temperature properties as well as free energies.

The empirical parameters in the force field, namely, the bonding parameters *k*_*i*_, *r*_*i*_^0^, θ_*j*_^0^, θ_*j*_^0^, γ_*n*_, *n*_*n*_, the partial charges *q*_*I*_, and the Lennard-Jones parameters ϵ_*IJ*_ and *σ*_*IJ*_, are determined using a wide range of strategies. These parameters will therefore be subject to uncertainties that will incur uncertainties in the sampling-based properties determined in molecular dynamics simulations using such classical force fields.

Brunken and Reiher^319^ proposed a system-specific parametrization of classical force fields of this form with respect to quantum-chemical reference data. For their parametrizations, they obtained uncertainty estimates by using the standard deviations from a *k*-fold cross-validation and from a separate Δ-ML model.

However, in contrast to machine learning interatomic potentials (see section 4.1.5), classical force fields are usually not parametrized to reproduce the potential energy surface from quantum-chemical calculations. Therefore, the uncertainty of force fields parameters cannot be determined directly, but has to be inferred from their impact on sampling-based properties. Such approaches will be discussed in section 4.2, in particular in section 4.2.1.

#### 4.1.5 Machine Learning Interatomic Potentials

Machine learning interatomic potentials (MLIPs) aim to model the potential energy surface *E*_0_^el^(***R***_1_, ***R***_2_, ...) of large systems at low computational cost with the accuracy of high-level *ab initio* calculations.^320,321^ In reality, these are often targeted to reproduce either static DFT or *ab initio* molecular dynamics simulations.^322,323^ Thus, MLIPs will inherit the flaws and errors of the reference data and will never be more accurate than them. Therefore, UQ in the context of MLIPs always aims at quantifying the uncertainty with respect to their reference method. Various forms and architectures of MLIPs have been proposed in recent years, and the most relevant ones in terms of this review and uncertainty quantification are briefly recapitulated in the following.

Neural network potentials (NNPs)—as the name suggests—use neural networks (NNs) to represent the potential energy of a system as an arbitrary analytical function of the atomic positions. Initiated by the work of Behler and Parrinello,^38^ various types of NNPs have since been developed, as comprehensively reviewed by Behler.^321^ An integral part of the training process of NNPs and many other MLIPs is active learning (AL), which refers to the iterative selection of the most informative data points (e.g., atomic configurations) to include in the training set, guided by uncertainty or model disagreement.^324^ This approach reduces the need for large, uniformly sampled datasets by focusing expensive reference (e.g., DFT) calculations on regions of configurational space where the model is least confident. Several strategies exist to guide this data selection process based on uncertainty quantification: Query-by-committee (QBC)^325^ uses an ensemble (committee) of models—typically neural networks—and quantifies uncertainty via their disagreement on a given input, e.g., by the variance or maximum deviation in predicted forces. If the ensemble variance is large for a given configuration, a reference calculation is performed and the new data is added to the training set. A key advantage of ensemble-based AL methods is the computational efficiency of their uncertainty quantification.^326^ While some authors have found that the predicted uncertainty and the actual error are not always linearly related,^327^ techniques to overcome this issue have been proposed.^328^ An alternative UQ approach is Gaussian process regression (GPR),^163,168^ where uncertainty is estimated analytically from a covariance (kernel) function that measures the similarity between unseen and previously observed data points. Another class of machine learning interatomic potentials is based on the atomic cluster expansion (ACE)^329–331^ introduced by Drautz and co-workers, a framework that expresses the potential energy as a linear combination of invariant basis functions constructed from atomic clusters. In the ACE approach, uncertainties can be estimated, for instance, using Bayesian linear regression, where the posterior distribution over model parameters naturally yields predictive variances. Fur further discussions of UQ for MLIPs, we refer to other reviews and perspectives.^70,84,168,320,332,333^

QBC approaches have for example been calibrated^334^ and applied to Raman spectroscopy,^335^ solvation of organic molecules^336^ as well as the radial distribution function and thermodynamic state functions of liquids^337^ by Ceriotti and co-workers. Their ensemble calibration^334^ is based on GPR and discussed in section 4.3.5. Isayev, Dral, and co-workers^338^ applied QBC to estimate uncertainties in the prediction of thermochemical properties with MLIPs. Recently, Kellner and Ceriotti^339^ introduced direct propagation of shallow ensembles, where the NN models of an ensemble share all but the last layer weights. Using datasets of DFT-computed properties for liquid water, lithium thiophosphate (Li_3_PS_4_), barium titanate (BaTiO_3_), and QM9 molecules, the authors demonstrate that their approach balances computational efficiency with reliable uncertainty estimation. Upon comparison against deep ensembles and conformal prediction methods, the approach improves predictive uncertainty without significant computational overhead. In this regard, conformal prediction can be used to estimate error bars for energy or force predictions, with formal statistical guarantees. Deep ensembles refer to ensembles of NNs, where each NN is augmented to also predict an uncertainty (e.g., the variance) for each input. These were investigated and compared to traditional committees by Carrete et al.^340^ They developed different MLIPs for the ionic liquid ethylammonium nitrate using both approaches and find that when using homogeneous training data (i.e., all data points generated at the same quality and/or method), deep ensembles do not show superiority compared to committees in terms of their uncertainty metrics. However, when training data from different sources was used, the deep ensemble could clearly discriminate between those sources. A different approach was chosen by Lin et al.,^341^ who focus on generating neural network (NN) based reactive potentials, specifically applied to H_2_ dissociation on Ag(111) and Ag(100) surfaces. They train an ensemble of NNs, then choose the two models with the lowest training errors, and from a newly defined uncertainty metric (the negative of squared difference surface) of these two models, determine points on the PES that should be subject to new *ab initio* reference data calculations. For their examples of hydrogen on silver surfaces, that approach worked reasonably well.

The dropout method was developed to overcome the computational costs of training a large committee of models required for good statistics. Dropout NNs are created from fully connected NNs by randomly removing the outgoing connections of some nodes in each layer, which is promising, because it has been shown that when using a variational inference approach,^342^ a dropout NN approximates a Bayesian NN thus enabling Bayesian UQ. Dropout-based and related techniques are best understood as pragmatic tools for probing predictive variability in ML models, rather than as comprehensive uncertainty quantification methods in the traditional UQ framework. Such approaches provide heuristic and approximate uncertainty estimates and should therefore be interpreted with appropriate caution, particularly with respect to overconfidence. The reliability of uncertainty estimates obtained from such methods should therefore be assessed using dedicated uncertainty validation techniques (cf. section 3.6) rather than inferred from the uncertainty model alone.

Wen and Tadmor^343^ used dropout NNPs in predicting mechanical properties of graphene. With a similar motivation, Zaverkin et al.^344^ use biased MD simulations to study the capability of MLIPs to sample the complex conformational space of alanine dipeptide and a flexible MOF. The authors use gradient-based uncertainty estimates, which they had developed in a previous work:^324^ that is, the gradient of the output with respect to the model parameters indicates how much the prediction changes when the parameters are perturbed. If the model’s prediction is highly sensitive to small changes in the weights, it implies that the prediction is likely to be uncertain. Then, the uncertainty is used to bias the MD potential to drive the simulation to previously unexplored (i.e., high-uncertainty) regions. It is found that this approach greatly accelerates the exploration of the conformational space and that the accuracy of the developed MLIPs is on par with selected ensemble-based methods, while at a notably reduced computational cost.

In the context of ACE-based MLIPs, Lysogorskiy et al.^345^ compared uncertainty estimation strategies based on ensemble learning and the D-optimality criterion. The latter selects the most informative atomic configurations by maximizing the determinant of the information matrix (constructed from the ACE basis functions), which corresponds to minimizing the overall uncertainty in the fitted ACE model parameters. The authors test the two strategies on structural properties of copper,^346^ MD simulations of water and structures of Li_4_ clusters. They finally conclude that both ensemble and D-optimality learning provide similar predictions, while the former is computationally more expensive due to the training of multiple models. Best et al.^347^ combine ACE potentials with conformal prediction for investigating structural and stress properties of bulk silicon. While most of the aforementioned works in this section explore various methods to determine the uncertainty of a model prediction in their active learning scheme, van der Oord et al.^348^ worked on hyperactive learning, an accelerated AL scheme and combine it with ACE potentials. Similar to the aforementioned work by Zaverkin et al.^344^ and a study by Kulichenko et al.,^349^ biased MD simulations are used to expedite the exploration of previously unseen parts of the phase space of the AlSi10 alloy and a polyethylene glycol polymer.

Other than that, Bartók and Kermode state that in the context of MLIPs, the use of “probabilistic learning methods such as Gaussian process regression (GPR) is currently under-exploited, because of the tendency of the predicted errors to overestimate the true error”.^350^ In a proof-of-concept work, they expand on GPR and develop MLIPs for argon dimers and trimers calculated using a CCSD­(T) reference. The authors observe significantly improved accuracy of predicted error estimates of the MLIPs when optimizing the hyperparameters of their GPR by maximizing the leave-one-out cross-validation likelihood. Botu et al.^351^ have employed kernel-ridge regression (KRR), which is conceptually related to GPR, in the sense that both rely on a kernel function to measure the similarity of data points, but KRR gives the mean prediction of GPR under Gaussian noise, while only GPR provides formal uncertainty estimates. The authors presented a general framework for deriving MLIPs, using the example of elementary aluminum. The key ingredients of their workflow is an UQ approach of deriving confidence intervals for the force field predictions by deriving an expression for the standard deviation of the predicted force error as a function of the discrepancy between the predicted and reference data fingerprints, which works well for the systems considered in their study.

#### 4.1.6 Multiscale Models

Multiscale models combine different computational methods to achieve a description of complex molecular systems and materials. There is a vast number of multiscale modeling strategies, which can broadly be categorized into two classes. First, in vertical multiscale models, accurate methods at smaller length and time scales are used to obtain parameters that are then used as input for lower-resolution methods which are able to reach larger length and time scales. This strategy is widespread in multiscale materials modeling. Here, uncertainty quantification can be performed by propagating an uncertainty of these parameters obtained at the smaller scale to the quantity of interest provided by the larger scale model. As the modeling of large-scale materials properties is beyond the scope of this review, we only refer to previous reviews on uncertainty quantification in multiscale materials modeling.^352–356^

Second, in horizontal multiscale models, computational methods of different accuracy are used to describe different parts of a complex molecular system. Usually, there is a particular region of interest, such as the active center in an enzyme, for which more accurate (and more computationally expensive) methods are used, while its environment is described using less accurate and computationally cheaper methods. The most prominent example of such a strategy are QM/MM methods.^357–359^ In addition, there is a plethora of QM/QM methods, in which different quantum-chemical methods are combined.^43,360–362^ Closely related are quantum-chemical fragmentation methods, in which a complex molecular system is split into smaller fragments, which are treated individually using the same quantum-chemical method.^363,364^

Even though all these methods have been developed with the goal of allowing for the prediction of a quantity of interest (e.g., the activation energy in an enzymatic reaction) with a certain required accuracy, while accepting larger uncertainties for parts of the system that are less relevant for this quantity of interest, there have been hardly any attempts to rigorously quantify these uncertainties.

##### Many-Body Expansion

The many-body expansion (MBE)^364^ is a prototypical fragmentation method, in which the total energy of a large system is approximated as

87 $$E_{\mathrm{tot}}=\underset{I}{\sum }E_{I}+\underset{I<J}{\sum }\Delta E_{IJ}+\underset{I<J<K}{\sum }\Delta E_{IJK}+...$$

where *E*_*I*_ is the energy of the *I*th fragment, Δ*E*_*IJ*_ = *E*_*IJ*_ – *E*_*I*_ – *E*_*J*_ is the interaction energy of the dimer made up of fragments *I* and *J*, Δ*E*_*IJK*_ is a trimer interaction energy, and so on. The sum is usually truncated at low order, i.e., after two-body or three-body terms.

Herbert and co-workers^365^ investigated the propagation of numerical errors in the MBE. Because the individual interaction energies in eq 87 are obtained as differences between total energies, their precision in floating point arithmetics is limited, and the numerical errors in the many small interaction energies can accumulate to substantial errors in the total energies, in particular for higher-order contributions.

Several studies have investigated the convergence of the MBE, in particular for molecular clusters, and have devised strategies for screening higher-order contributions, i.e., to neglect selected terms in eq 87. Such strategies are—at least implicitly—based on an estimation of the size of these individual higher-order contributions and of the uncertainty in the total energy resulting from their neglect. The simplest strategy estimates the relevance of individual contributions from the distance between the involved fragments (distance-based screening).^366–368^ A more sophisticated estimate relying on electrostatic dipole–dipole interactions, which also takes the orientation of the different fragments into account, was developed in ref 369. Finally, the individual contributions can be estimated by calculating them at a lower level of theory, such as a polarizable force-field or semiempirical quantum mechanics (energy-based screening).^370,371^

In the many-body expanded full-CI (MBE-FCI) method,^372^ an expansion akin to eq 87 is applied to the correlation energy, with the correlated orbitals used as “fragments”. Greiner et al.^373^ developed an error estimation scheme for the MBE-FCI method. First, for each orbital it establishes an estimate of the maximum magnitude of higher-order contributions by a fit to the lower-order contributions. Second, for groups of orbitals it determines the distribution of the contributions at a certain order by Monte Carlo sampling. The latter makes it possible to exploit the fact that many contributions of opposite signs will cancel. These error estimates are used to truncate the MBE-FCI expansion, while rigorously controlling the maximum error in the resulting total energy.

##### QM/MM Models

In QM/MM models, the system is split into a region of interest, which is treated using quantum-chemical methods (QM region), and its environment, which is treated using a classical force field (MM region). Despite the long history of QM/MM methods^357–359^ and their broad application, in particular for studying enzyme catalysis,^374–378^ the accuracy of such models is hard to assess. This was recently highlighted by Giudetti et al.,^379^ who found that the QM/MM reaction energies obtained with implementations in different software packages can show very large differences, even if the same structural models and QM/MM methods are used. These difference could partly be traced back to minor differences in technical settings, which propagate to substantial differences in the calculated reaction energies. This underlines the need for systematic uncertainty quantification and error control in QM/MM calculations.

This becomes even more pressing when trying to construct suitable QM/MM models for a specific system of interest, which involves numerous choices by the computational scientist.^376,377,380^ Most relevant is the choice of the QM region (i.e., selecting which parts of the system are treated quantum-chemically). Systematic studies have demonstrated that the convergence with respect to the size of the QM region is slow and in many cases not monotonic.^381–387^

Several schemes have been developed to assist or automate the choice of the QM region in biomolecular QM/MM calculations (for a review, see ref 388). The first group is based on some form of sensitivity analysis, in which the effect of the change in some parameter related to each amino acid on the property of interest (usually an energy difference) is used to select those with the largest sensitivity for inclusion in the QM region. In the charge-deletion analysis,^389,390^ the effect of deleting the MM charges of individual amino acids is used. In the point-charge variation analysis,^391,392^ this is extended by using a local sensitivity analysis with respect to MM charges.

A second group of approaches uses different descriptors to predict the importance of individual amino acids in the QM region, often based on calculations using large QM regions. The charge-shift^385^ and Fukui shift analysis^393^ compare the change in the charges between the apo and the holo form. Brunken and Reiher^394^ compare the energy gradient between large and small QM regions. Cisnero and co-workers^395^ proposed an approach based on protein sequence similarity, and in ref 396, protein network centralities were explored as descriptors in the construction of the QM region.

### 4.2 Uncertainties in Trajectories and Related Properties

While the works discussed in section 4.1 pertained to solving the stationary (time-independent) Schrödinger equation or approximations thereof, this section is devoted to works exploring the dynamics of matter i.e., solving time-dependent problems. The most popular method of dynamically traversing potential energy landscapes are molecular dynamics (MD) simulations, which propagate an ensemble of particles in time by solving Newton’s equations of motion. Given an ensemble of *N* particles with masses *m*_*i*_ and nuclear positions ***R***_*i*_, the force ***F***_*i*_ acting on particle *i* is given by

88 $$F_{i}\left(t\right)=m_{i}\frac{d^{2}R_{i}\left(t\right)}{dt^{2}}=-\nabla_{R_{i}}V\left(R_{1},...,R_{N}\right)$$

where *t* is the time, and *V* is the potential that explicitly depends on the positions of all *N* particles. We note that for classical force-field-based MD simulations, *V* is usually equivalent to *E*_FF_ from eq 86. Unfortunately, the above equation cannot be solved analytically for *N* > 2 and is thus numerically integrated in small intervals δ*t*, typically 0.5 to 1 fs. During a simulation, the phase space Γ­(*t*), a function of the positions and velocities, is sampled, and these quantities are stored as a trajectory.

For completeness, we also mention the concept of *shadowing*,^397^ which has gained attention in the simulation community recently.^398–400^ It is known that trajectories with slightly different initial conditions (positions or velocities) diverge exponentially in phase space.^397^ As MD simulations rely on numerical integration for solving Newton’s equations of motion, the conservation of energy, and maintaining the “true” path in phase space are critical for producing reliable predictions. It was found that modern symplectic integrators from the Verlet family usually produce trajectories that shadow the true trajectory, meaning that the simulated trajectory stays “close” in phase space to the true one.

It is evident that the complexity of the integration is largely dependent on **V**—in fact, different flavors of MD simulation are distinguished by certain classes of functional forms **V** can adopt. Among the most common forms, force-field-based MD simulations and *ab initio* MD (AIMD) simulations have evolved. The former typically rely on a pairwise-additive and computationally simple force field (see section 4.1.4), thus allowing for simulations involving several thousand atoms and time scales up to the μs range.^401,402^ AIMD simulations feature a more complex potential, mostly by means of DFT (see section 4.1.2), and therefore, come with drastically increased computational cost, but provide information on electronic structure of a dynamic system, enabling, for example, the computation of vibrational spectra from time-correlation functions of the molecular dipole moments.^403–405^ For a more detailed introduction, the reader is referred to refs 96, 97, 318, 406, and 407.

MD simulations generally suffer from both aleatoric and epistemic uncertainties (refer to section 2.1), the former primarily being caused by incomplete sampling due to finite computational resources, and the latter arising through the lack of information on the physical/chemical model.

Aleatoric uncertainties can often be compensated for by investing more computing time and performing more or longer simulations, and applying enhanced sampling techniques. The simulation community has become increasingly aware of this matter, and efforts to promote standardized simulation protocols for reproducible simulations have been made (see ref 196 and references therein). Specifically, Coveney and co-workers^408^ emphasize repeatedly that MD is intrinsically chaotic and therefore, ensemble simulation methods are needed independent of the duration that is carried out, because MD is extremely sensitive to the initial conditions, “making accurate predictions impossible and one-off observations largely unreproducible even though their underlying dynamics is deterministic”. Consequently, it has been recommended by several researchers^65,409,410^ to increase the statistical robustness of MD simulation studies by performing an ensemble of simulations (also known as replica simulations), and computing properties as ensemble averages including meaningful uncertainties.

Tackling epistemic uncertainties for MD simulations typically involves either (i) improving the physical model, e.g., by adjusting the functional form of the force field, or (ii) updating the parameters of the physical model to reproduce certain reference data more accurately, or (iii) improving the chemical model by including more molecules or a considering a different representation of the system of interest. While measures to treat aleatoric uncertainties sometimes appear straightforward, suitable paths to treating epistemic uncertainties may not always appear obvious. However, various UQ techniques have been applied to both sources of uncertainties in the context of MD simulations, and are reviewed in this section.

At the same time, epistemic uncertainties in MD simulations may also arise from issues of code correctness and model adequacy. In the terminology commonly used in VVUQ frameworks,^171^ verification refers to ensuring that the numerical implementation correctly solves the underlying equations of the chosen model, whereas validation concerns the extent to which the selected model faithfully represents the physical system of interest. While verification and validation are closely related to epistemic uncertainty, they address different questions than UQ itself and are therefore not treated as primary topics in this section.

Besides MD simulations, Monte Carlo (MC) methods constitute the second big branch of sampling-based simulation methods with a widespread use in *in silico* chemistry. In an MC simulation of an ensemble of particles with a given configuration (geometry), a random step in phase space is proposed by randomly moving one (or possibly more) particles. The energy for the new configuration is calculated and the move to that new configuration will be accepted with a certain probability, typically involving the negative exponential of the energy. Otherwise, a new random step in the phase space is proposed, and the procedure is repeated. Recent advances in this field concern hybrid Monte Carlo,^124,411^ kinetic Monte Carlo,^412,413^ and quantum Monte Carlo methods.^414–416^ It is important to note that similar to MD, MC simulations are inherently stochastic too, as detailed in section 3.4.1 and refs 96 and 97. So far, the literature on UQ for MC simulations remains sparse.

#### 4.2.1 Simulation Parameters

Tracing and propagating the uncertainty from input to output (commonly referred to as sensitivity analysis, section 3.3) is an important challenge in the general field of computational simulations and consequently, various approaches to tackle this challenge have been reported. Applied to molecular dynamics simulations, the aforementioned mostly refers to investigating the propagation of uncertainties in the simulation input parameters to the QoIs predicted based on the simulation, such as density, diffusion coefficient, or radial distribution function. Uncertainties in the input parameters are mostly investigated with a focus on (i) force field parameters (bond lengths, partial charges, LJ parameters) or (ii) system parameters (e.g., number of molecules, temperature, simulation box size). For completeness, we will also distinguish *local* sensitivity analysis (section 3.3.2), that considers functional derivates and polynomial approximations thereof for quantifying uncertainties (also known as “forward propagation”), from *global* sensitivity analysis (section 3.3.1), where Sobol’ (or other types of) indices are computed to derive an ordering, indicating how strongly each input parameter affects the output. Most works discussed in this review fall into the first category, likely because the second often is computationally much more demanding. Given that UQ for MD simulations is an emerging, and not yet fully established field, it is understandable that existing works on (local or global) sensitivity analysis for MD simulations mainly address either systems with small molecules or well-defined, simple materials, featuring manageably complicated functional forms and low-dimensional parameter spaces.

We will first discuss works with an application to molecular systems. Rizzi et al.^417,418^ performed a local sensitivity analysis on force field parameters in MD simulations, at the example of the TIP4P water model. By combining polynomial chaos expansions with Bayesian inference (see sections 3.2.2 and 3.4.4), they found that the force field parameters are scarcely sensitive to each other while being very sensitive toward specific observables of the system, such as density and diffusivity. After that, they conducted two studies on the ion transport of NaCl in aqueous solution through silica nanopores. While the first work^419^ focused on examining the influence of the pore diameter and the gating charge on the ionic flux, the authors investigated uncertainty in the potential parameters and their impact on the flux in the second work.^420^ Molinero and co-workers^43,421,422^ followed a similar approach, employing polynomial chaos expansions in deriving parameters for a coarse-grained force field model for simulating hydrated anion-exchange membranes and fuel cell membranes with controlled water uptake.

Peerless et al.^423^ studied how the uncertainty associated with partial atomic charges in MD simulations impacts diffusion, density and solubility in the bulk phase, using the example of liquid acetonitrile, described by the General Amber Force Field (GAFF).^424^ The formulation and application of a Gaussian process regression model (section 3.5.2), demonstrates a notable speed-up in generating additional sampling and thus, achieves rapid predictions of local sensitivities of bulk phase properties. In the context of high-performance computing, Angelikopoulos et al.^425^ introduced a Bayesian probabilistic framework designed to quantify and propagate the uncertainty in parameters of force fields for classical molecular dynamics simulations. At the example of liquid argon, it is demonstrated how uncertainties in the parameters of the intermolecular interaction potential (in this case, the Lennard-Jones potential) are propagated in MD simulations, resulting in confidence intervals for the predicted transport quantities (e.g., diffusion coefficient and viscosity). The authors then build on their previous work and systematically assess the uncertainties of properties predicted in MD simulations of nanofluidic water transport and confined water/carbon interfaces.^426^

Messerly et al.^427^ investigated the transferability of Mie λ-6 force fields for alkanes, derived at standard conditions, to very high temperatures and pressures. By utilizing Bayesian inference, the authors demonstrated that, for the systems investigated, there is no valid combination of Mie potential parameters that can predict liquid–vapor equilibrium properties (density and pressure) at harsh thermodynamic conditions. From the authors’ viewpoint, that highlights the demand for more accurate potentials in applications with industrial interest. In a follow-up study,^428^ the authors further explored finding the optimal force field parameters to describe the aforementioned vapor–liquid equilibrium properties. Two types of surrogate models were explored in sampling the parameter space: One model, multistate Bennet acceptance ratio was found to be promising when exploring remote regions of the parameter space, while the other, pair correlation function rescaling proves to be helpful in the more localized regime. Raabe et al.^429^ used Gaussian process regression (section 3.5.2) for deriving force field parameters for *trans*-1,2-dichloroethene, based on its vapor–liquid equilibrium properties.

On a related note, Madin et al.^430^ examined three types of Lennard-Jones force fields with optional additional quadrupole terms and increasing complexity, targeting at density, saturated vapor pressure and surface tension of Br_2_, F_2_, N_2_, O_2_, C_2_H_2_, C_2_H_4_, C_2_H_6_, and C_2_F_4_. By using Bayesian inference for selecting the type of force field, a model’s complexity and computational effort is weighed against the accuracy and precision of its predictions. In that study, it turned out that an increased model complexity compared to the common Lennard-Jones potential is only worthwhile in a few cases. With the goal of finding the optimal parameters for Mie λ-6 force field for liquid neon, Shanks et al.^431^ presented local Gaussian process surrogate models, trained on X-ray/neutron diffraction scattering data. They find that their local surrogate models are much faster in computing the force field parameters because of a better scaling with the number of independent variables compared to standard Gaussian processes. Dutta et al.^432^ studied the Bayesian calibration of force field-parameters of water and helium, assuming their uncertainty not to be Gaussian. In particular, they used approximate Bayesian computation, a likelihood-free inference scheme, and its implementation for HPC systems. The application of their method is presented using datasets from neutron and X-ray diffraction measurements and MD simulations. As the proposed method provides access to the entire posterior distribution, uncertainty quantification of the model predictions is possible and thereby, can in principle, calibrate force fields from any type of structural or dynamic property data.

Turning to solid-state applications, Tran and Wang^433^ introduce a reliable molecular dynamics (R-MD) framework to assess the output of MD simulations, given the input uncertainty in the intermolecular potential and/or the parameters. At the heart of R-MD, there is the formulation of input uncertainty in the potential as intervals, and as a consequence, positions and velocities are interval-valued, too. In a follow-up work,^434^ the authors describe four different schemes of uncertainty propagation in the framework of R-MD and apply their developments to predict the tensile uniaxial deformation of aluminum single crystals, described by an embedded atom method (EAM) potential.^435^ Similarly, Dhaliwal et al.^436^ also studied the local sensitivity of the parameters of EAM potentials of face-centered cubic aluminum, using a Bayesian statistical framework, while Vohra et al.^437^ used nonequilibrium molecular dynamics (NEMD) simulations to capture thermal transport in silicon and performed a local sensitivity analysis on the potential parameters. By deriving a surrogate model for the NEMD simulations from a reduced-order polynomial chaos expansion (section 3.4.4), the authors could quantify which parameters in the employed Stillinger–Weber potential^438^ contribute to the uncertainty of predicted bulk thermal conductivity in Si.

Studying solid nickel, Longbottom and Brommer^439^ implemented a Bayesian framework for propagating uncertainties in three types of potentials (LJ, Morse, and EAM), derived from DFT reference data, to simulated lattice constants, elastic moduli and thermal expansion coefficients. The work by Kurniawan et al.^440^ investigates classical interatomic potentials such as the Lennard-Jones, Morse, or Stillinger–Weber potential for several metals and materials from a conceptual viewpoint and shows that these are mostly sloppy, i.e., insensitive to concerted changes in certain sets of parameters.

Lastly, a few applications of local sensitivity analysis with coarse-grained MD simulations are known, mainly by Müller-Plathe and co-workers. They developed a coarse-grained molecular dynamics-finite element coupling approach to investigate the mechanical behaviors of polymers. That approach partitions the system under investigation into an MD region and a continuum (finite element) region, requiring several technical input parameters, whose values cannot always be deduced based on physical principles. Therefore, in their first work,^441^ the authors investigated polystyrene and performed a local sensitivity analysis of the polymer structure (density, radius of gyration, end-to-end distance and radial distribution function) on these input parameters. They find that the simulation technique is generally robust with the polymer properties being only weakly dependent on the simulation parameters (number of anchor points, force constant between polymer and anchor points and the size of the molecular dynamics domain). This first work, however, exclusively focused on the MD region and did not consider the continuum region in the UQ. In a follow-up work,^442^ the authors then also considered the continuum region in their UQ treatment and identify trustworthy ranges for the simulation parameters.

We also reference a rather mathematical description on sensitivity analysis of observables from Langevin dynamics simulations.^443^ Further works involving sensitivity analysis in the context of free energy calculations are discussed in section 4.2.4.

#### 4.2.2 Time Correlation Functions

In molecular dynamics simulations, dynamical quantities, such as self-diffusion coefficients, viscosities as well as thermal and ionic conductivities, are accessible via Green–Kubo type correlation functions. Those, however, can be subject to large uncertainties caused by inadequate sampling. At this point, replica trajectories in conjunction with bootstrapping (section 3.4.2) are suitable to increase the statistical robustness of the calculations. Fischer et al.^444^ developed a protocol for bootstrapping correlation functions from simulations of ethane, propane, and dimethyl ether in conjunction with the time decomposition method of Maginn and co-workers.^445^ Desbiens et al.^446^ also apply bootstrapping to the current and velocity autocorrelation function, highlighting that the increased sampling can compensate for shorter simulation times or smaller number of replica simulations.

Kirchner, Frömbgen, and co-workers provided two tutorials on how to reduce the uncertainty in the simulation and analysis of ionic liquid trajectories,^410,447^ with a focus on dynamic properties, such as self-diffusion coefficients and ionic conductivities. These quantities involve calculating the (individual or collective) mean squared displacement (MSD) of particles, based on correlation functions of the particles’ positions, and performing a subsequent linear regression of the linear regime of the MSD. Rigorously identifying that linear regime is not a straightforward task, especially in the case of the collective MSD which is used for computing the ionic conductivity, due to poor sampling. In avoiding “eyeball statistics”, Frömbgen et al.^410^ proposed a systematical procedure for identifying linear regimes in MSD data, by exploiting the fact that the slope of log­(MSD) has to equal unity within the linear regime. This procedure was also used in a recent work by Frömbgen et al.^448^ that implemented a multifidelity Monte Carlo strategy (section 3.4.5) for simulating self-diffusion coefficients of liquid water from MD simulations. By exploiting the dependence of the self-diffusion coefficient on the simulation box size, the authors constructed a multifidelity model hierarchy based on differently sized simulation boxes. It was shown that for cubic simulation boxes, in terms of the mean squared error of the predicted self-diffusion coefficient as a function of the computational budget, any combination of models is superior to solely using a single high-fidelity model.

A multifidelity Gaussian process approach (see sections 3.4.5 and 3.5.2) to calculating shear viscosities of small to medium-sized liquid aliphatic alkanes and alcohols, based on the autocorrelation function of the stress tensor, was proposed by Fleck et al.^449^ In that work, a few high-fidelity experimental data points is combined with a larger number of low-fidelity data points from MD simulations to train a GP model and predict shear viscosities at various thermodynamic state points (temperature, pressure, density) with high accuracy.

#### 4.2.3 System Size, Time, and Length Scale: Enhanced Sampling and Reweighting Methods

The predictions from molecular simulations are often limited in terms of sampling relevant sizes or times of a particular system at interest, occurring frequently in the field of biomolecular simulation. UQ methods targeting such applications are addressed in the following. It should be noted that, as the section on free energy calculations partially overlaps with this section, works relevant to the former topic are discussed in section 4.2.4.

In scenarios where only a few samples of QoIs can be calculated for computational reasons, such that the measured probability distribution does not allow for computing meaningful averages and standard deviations, Bayesian bootstrapping can be a promising approach. Mostofian and Zuckerman^450^ apply this method to study the rate constants of protein folding, simulated by means of MD, and conclude that it is well-suited method for high log-variance datasets. Xia and Wei^451^ also address the challenge of modeling protein folding, specifically by introducing molecular nonlinear dynamics, an approach in which the atoms are represented by chaotic oscillators. The authors then show that folding a protein reduces the chaoticity of the system and finally, build on chaos to devise an algorithm for thermal protein uncertainty quantification. Russo et al.^452^ proposed a trajectory reweighting scheme allowing for prediction of various observables from sets of short, unbiased trajectories, and applied it to a 1 μs tryptophan cage folding trajectory.

#### 4.2.4 Free Energy Calculations

Free energy calculations are at the heart of understanding various kinds of chemical processes, for example reactions, protein–ligand binding, solvation of particles, and wetting at liquid/solid interfaces. The principles of free energy calculations are rooted in statistical mechanics and derived from the density of states or the partition function. In practice, however, *in silico* chemistry does not seek to calculate absolute free energy of a system, but the *difference* in free energy of a system in two states, where 0 is the initial or reference state, and 1 is the final or target state. Chemically, the transition of a system from state 0 to 1 may, for example, correspond to (i) transferring a molecule from the gas phase into a solvent, enabling the calculation of the solvation free energy, or (ii) bringing a protein in touch with a ligand, giving rise to the binding free energy. For a deeper introduction to this matter, we refer the reader to the comprehensive book by Chipot and Pohorille,^453^ or existing reviews.^454–461^ From a bird’s-eye perspective, the most prominent approaches to calculating free energies are based on the free energy perturbation method,^462^ thermodynamic integration,^463^ or probability distributions and histograms^464,465^ and will be briefly summarized in the following:

Free energy perturbation (FEP) relates the free energy difference between two states to the ensemble average of the exponential of their energy difference. A key advantage of the method is that the energy of both states 0 and 1 is solely evaluated in one of the phase spaces, conventionally in that of 0, and thus, renders the method computationally efficient. It is, however, only suitable when the two states are very similar (i.e., their phase spaces overlap strongly), and becomes unreliable when this overlap is poor, as the exponential averaging introduces large statistical noise. To correct for a poor overlap in phase space between two states, a number of intermediate states can be introduced, ensuring sufficient overlap between adjacent states.

Thermodynamic integration (TI) circumvents the overlap issue by introducing a continuous coupling parameter λ which smoothly transforms the system from state 0 to state 1 via a hybrid potential *V*. The free energy difference Δ*F* is computed by integrating the average of the derivative of the potential energy with respect to the coupling parameter:

89 $$\Delta F=\int_{0}^{1}{\left\langle \frac{\partial V\left(\lambda \right)}{\partial \lambda }\right\rangle }_{\lambda }\;d\lambda$$

Note that similar to the FEP method, a sufficient phase space overlap between the states or the consideration of intermediate states is required. TI is more robust than FEP across a broader range of transformations but requires careful choice and spacing of coupling points to ensure smooth convergence and accurate numerical integration. In this context, *alchemical* free energy calculations should be mentioned, that, referring back to ancient alchemists who sought to transform lead into gold, involve transforming (mutating) a chemical species into an alternate one. Thus, the difference in the potential stems from the different chemical species in state 0 and 1. Note that the majority of works referenced in the following is based on TI.

Histogram-based methods (or reweighting methods), including umbrella sampling and the weighted histogram analysis method, are particularly useful for systems involving slow transitions along a well-defined reaction coordinate. These methods apply biasing potentials to enhance sampling in poorly explored regions of phase space, generating overlapping histograms of sampled configurations across several windows. While offering a powerful tool, such methods require prior knowledge of a suitable reaction coordinate and can be computationally demanding to achieve sufficient overlap. In practice, the multistate Bennet acceptance ratio (MBAR) estimator by Shirts and Chodera^466^ is often used in works that address UQ in the context of histogram methods.

It should be noted that, when discussing UQ in terms of free energy calculations, existing works can be roughly allocated to two main categories, namely UQ with respect to (i) the free energy method itself (e.g., those laid out above) and (ii) the sampling of the phase space, which is inevitable to accurate free energy predictions. The second category clearly has an overlap with section 4.2.3, and thus, all works that are relevant to free energy calculations are discussed below.

Many studies with respect to the uncertainty quantification in free energy calculations, especially on alchemical free energies, were published by Coveney and co-workers.^408,467–473^ In ref 467, Bhati et al. introduce UQ for TI-based alchemical free energy calculations by a method called thermodynamic integration with enhanced sampling (TIES). In traditional TI approaches, the derivative of the potential (eq 89) is typically obtained from a single MD simulation for each discrete window of the coupling parameter, while in TIES, an ensemble of MDs is performed for each window. This procedure has two advantages: first, by performing multiple replica simulations, sampling of the phase space is greatly enhanced and second, the ensemble provides access to statistical quantities and hence, UQ.^408,469^ The authors have found that *∂V*/*∂λ* behaves like a Gaussian random variable and therefore, treat the integral as a stochastic one, which is solved numerically and provides a variance of Δ*F* based on the bootstrapped standard error in each λ window. Combining TIES with several enhanced sampling methods,^474,475^ Bhati et al.^468^ calculate free energies for seven protein–ligand pairs, and (i) find that the accuracy of the results is improved by an ensemble of replica simulations compared to performing a single very long simulation, while (ii) they investigate the performance of the enhanced sampling schemes with respect to their computational effort. Recently, an updated TIES version was introduced by Bieniek et al.,^470^ that uses a new protocol to superimpose the two transformed species (often ligands) in alchemical free energy calculations. That, for example, allows for aromatic rings to be partially superimposed, which reduces the size of the alchemical region and hence, the TIES error, while improving the precision of the predicted free energies. For completeness, we also mention the work by Wade et al.^471^ and Bhati et al.^472^ here, which apply the aforementioned UQ methods for alchemical free energies two large benchmark studies on ligand transformations using different MD simulation packages in conjunction with TI and FEP methods.

Further, Vassaux et al.^469^ performed a large-scale study on simulating the binding free energy of the bromodomain-containing protein 4 and the tetrahydroquinoline ligand with approximated continuum solvent.^476^ The authors investigated, how uniformly distributed uncertainty in a set of 14 input parameters, including the simulation temperature, pressure and duration of equilibration/production runs, is propagated through MD simulations to estimate the binding free energy of the protein–ligand pair. Additionally, the influence of the random velocity seeds is investigated by means of replica simulations. Uncertainty is propagated through the simulations by applying a dimension-adaptive version of stochastic collocation^139,145^ (refer to section 3.4.4). It is found that the variance in the predicted binding free energies is primarily dominated by six parameters, including temperature, box size and barostat settings. Also, the authors show that the uncertainty is “damped” during the simulation, meaning that the variation of the computed binding free energies around the mean are smaller than for the input parameters. In a follow-up work, Edeling et al.^473^ conducted a conceptually related sensitivity analysis, investigating the influence of uncertainties in force field parameters on the stiffness of a polymer material and the binding free energies of two protein–ligand systems, but propagating uncertainties by a Gaussian process method. Note that studies on sensitivity analysis of MD parameters not related to free energies are discussed in section 4.2.1.

On the notion of using histogram methods for free energy calculations, the works by Schieber et al.^477^ and Bauer and Gross^478^ investigate phase diagrams of solid benzene as well as argon, methanol, and water, respectively. Both studies employ the MBAR histogram method^466^ and estimate uncertainties in their predictions of the phase coexistence lines using bootstrapping^478^ (section 3.4.2) or standard error propagation.

#### 4.2.5 Coarse-Grained Simulations

The importance of coarse-grained (CG) simulations has been discussed by Noid in a recent perspective,^479^ where the author explains that “by representing systems in reduced detail, CG models provide the necessary computational efficiency for simulating length- and time-scales that remain far beyond the scope of conventional atomically detailed simulations”. As the aforementioned “reduced detail” often come along with approximations and (over)­simplifications, CG simulations are highly concerned with uncertainty quantification.

Molinero and co-workers have worked on UQ for CG simulations of anion exchange membranes^421,422^ (see section 4.2.1).

Naturally, CG force fields for water are in high demand as well, and thus, it comes as no surprise that these have been investigated by means of UQ. Zavadlav et al.^480^ developed a Bayesian framework for the data-driven selection of CG water models for a given application. Most importantly, the authors find that flexible water models do not provide improved accuracy, and consequently, rigid water models are recommended for computational efficiency. Also, the charge distribution within the model is found to be another key quantity, with more complex charge models delivering the best accuracy.

A central issue, not only in the CG community but also in the general field of force-field-based modeling, is transferability, i.e., the reusability of force fields beyond the specific chemical system for which they were developed. In their methodological work, Patrone et al.^481^ approach this issue for CG simulations specifically by deriving an iterative Bayesian correction algorithm to recalibrate the forces obtained through force-matching against thermodynamic state points.

### 4.3 Uncertainties in Static Properties and in Electronic Structure Applications

#### 4.3.1 Chemical Reaction Networks

A chemical reaction network is a set of interconnected chemical reactions that describe how different species transform into one another. It represents the overall reaction system as a network of reactants, products, and intermediates linked by elementary steps.

While handling prediction errors in species-specific properties is a challenge in itself, the situation becomes considerably more complex when the target property depends on a network of interconnected species. These species are coupled through elementary reaction steps, meaning that an inaccuracy in any activation energy across the network may affect the predicted concentration of the desired product over time. This effect can be substantial given that the concentration is a function of rate constants, each of which depends *exponentially* on its associated energy barrier. Thus, even minor deviations in activation energies can cascade through the reaction network, leading to substantial discrepancies in product concentrations. On the other hand, uncertainties in activation energies of different reaction steps are often correlated, in particular if these energies are obtained from quantum-chemical calculations.^482^

Reiher and co-workers demonstrated this cascading behavior for a small model network of the formose reaction (6 species, 10 elementary steps).^284^ By generating an ensemble of long-range-corrected PBE0 functionals via Bayesian statistics (cf. section 4.1.2 and ref 277, where BEEFs were combined with reaction networks to study dry reforming of methane in the context of heterogeneous catalysis), distributions—instead of single values—of activation energies and hence rate constants were obtained. The resulting species concentrations span up to 23 orders of magnitude in time. This early study on uncertainty quantification for chemical reactions demonstrates how critically sensitive network properties, such as time-dependent concentrations, can be to species-specific energy uncertainties.

In a follow-up study,^308^ Proppe and Reiher addressed an issue that was later^29^ summarized as follows: “The exquisite details that [network] exploration algorithms can generate for any chemical process raise the question of reliability as, in general, no or very little experimental or theoretical reference data will be available. Consequently, uncertainty quantification will become a crucial part of the whole exploration process.” To still demonstrate the effect of uncertainty-equipped activation energies for significantly larger reaction networks, KiNetX and the helper tool AutoNetGen were developed.^308^ The latter generates artificial, chemistry-mimicking reaction networks and equips each node (species) of a network with an ensemble of activation energies sampled from a predefined covariance matrix. Each diagonal element of the covariance matrix represents the variance of the corresponding energy barrier, while each off-diagonal element constitutes the correlation between a pair of activation energies.

Subsequently, KiNetX propagates this energy uncertainty by solving the underlying ensemble of kinetic models and estimates the kinetic relevance of each species based on its maximum rate of formation. The software then reduces the reaction network by identifying and eliminating kinetically irrelevant vertices and edges through a systematic hierarchy of flux analyses,^483^ thus achieving a more compact network representation of the reaction mechanism. Eventually, KiNetX performs a sensitivity analysis to distinguish between model parameters (here, activation energies) that significantly impact the outcomes (here, time-dependent species concentrations) and those that do not. This classification is particularly valuable as it helps in determining the quality of energy barriers obtained from efficient, semiaccurate quantum-chemical methods. If the uncertainty in the concentrations of certain species proves too large to make reliable conclusions about specific aspects of the reaction network, the result of the sensitivity analysis will highlight which activation energies are most critical. These critical parameters can then be re-evaluated using a more sophisticated electronic-structure method, ensuring a higher level of accuracy where needed.

In an actual exploration scenario, unlike the artificial networks generated by AutoNetGen, one would leverage uncertainty from the very beginning to guide the exploration process. So far, the discussion has focused on uncertainty in kinetic parameters, but when exploring unknown reaction networks, this uncertainty plays an even more central role. Rather than applying it retroactively, uncertainty actively informs the exploration, helping to prioritize which reactions and species to investigate further. This approach ensures that the exploration is both efficient and comprehensive, reducing the risk of overlooking critical pathways due to minor errors in activation energies.

This method has been applied in studies using the Reaction Mechanism Generator (RMG) software,^484^ where the propagation of uncertainty through reaction networks was demonstrated for carbon dioxide methanation on Ni(111)^485,486^ and exhaust gas oxidation on Pt(111).^281^ By analyzing ensembles of reaction networks, these studies quantified how uncertainty in activation energies can affect the entire network, ultimately influencing predicted product concentrations. Such analyses highlight the necessity of incorporating uncertainty at every stage of the exploration process, ensuring a more reliable understanding of the network’s behavior.

While the ensembles of the RMG-generated networks were obtained from a rule-based approach, which requires well-established reaction families and predefined graph rules, the Kinetics-Interlaced Exploration Algorithm (KIEA)^487,488^ and Yet Another Kinetics Solver (YAKS)^489^ methods circumvent these limitations by utilizing exclusively first-principles-based methods for the network exploration.

All of the above examples were based on the implicit assumption of mass action and, hence, reaction networks with deterministic kinetics. When stochastic effects are taken into account, the complexity of uncertainty quantification increases significantly. In reaction networks where stochastic kinetics is more appropriate, uncertainty arises not only from kinetic parameters but also from the inherent randomness of reaction events. This shift requires methods that can handle both types of uncertainty simultaneously. For example, Navarro Jimenez et al.^490^ developed a global sensitivity analysis approach that effectively disentangles these different sources of variability, offering deeper insight into the system’s behavior under stochastic conditions.

Understanding variability in reaction networks with stochastic kinetics requires not only accurate models but also carefully designed experiments that can maximize the information gained about the system. Ruess and co-workers^491^ developed a framework based on Fisher information^492^ to optimize experimental setups for identifying key system parameters. By leveraging the inherent stochasticity in reaction rates, their approach provides a means to quantify and reduce uncertainty more effectively. This method underscores the importance of experimental design in stochastic systems, ensuring that the variability in system behavior is accurately captured and used to refine model predictions. Such strategies can be invaluable in cases where stochastic noise dominates.

#### 4.3.2 Reactivity Parameters

Instead of focusing on entire reaction networks, the quantification of uncertainties can also be performed at the level of individual elementary reactions. Such uncertainties in individual rate constants can later be propagated through a network of these elementary reactions.

Particularly suitable for such quantification are the highly accurate experimental rate constants from Mayr’s laboratory, which have been measured and documented over the past decades for thousands of electrophile–nucleophile reactions. To enable efficient estimation for arbitrary combinations of reaction partners, in 1994 Mayr and Patz developed a three-parameter equation:

90 $$\log k=s_{N}\left(N+E\right)$$

where *E* denotes the electrophilicity and *N* the nucleophilicity, while *s*_N_ is an additional nucleophile-specific sensitivity factor. The two nucleophilic parameters are also solvent-dependent. The resulting rate constants are defined at a reference temperature of 20 °C.

In a 2022 study, Proppe and Kircher^493^ were the first to determine uncertainties for such reactivity parameters derived from experimental results, which, through propagation, enable the determination of uncertainties in the corresponding rate constants. In a 2023 review by Vahl and Proppe,^494^ various strategies were presented for estimating such reactivity parameters using quantum-chemical calculations. After Proppe and co-workers demonstrated in 2024 ^495^ how machine learning can be employed to generate quantum-chemically derived reactivity parameters in a high-throughput manner, they applied UQ for the first time in 2025 ^496^ to determine the uncertainty of a reactivity parameter, namely, the electrophilicity of carbon dioxide.

#### 4.3.3 Theoretical Spectroscopy

The calculation of molecular spectra and of spectroscopic properties is another important application of *in silico* chemistry, in particular of quantum-chemical methods. In many cases, the comparison between computations and experiment can serve as a tool for assigning spectral features and for elucidating molecular structures. This makes it even more pressing to quantify the uncertainties in such calculations in order to confidently draw such conclusions.

While the sources of uncertainties in the ground-state energies from quantum-chemical calculations that were discussed in section 4.1 also apply to quantum-chemical calculations of spectra, the latter usually requires additional steps that can introduce uncertainties. Some of these will be discussed in the following for different types of spectroscopy.

##### Structural Sensitivity of Calculated Spectra

Like all quantities of interest in computational chemistry, calculated spectra are subject to many sources of uncertainty. In the quantum-chemical calculation of molecular spectra, one important source of uncertainty is the choice of the molecular structure that is used in these calculations. This is particularly relevant if the comparison of experimental and calculated spectra is used to infer structural parameters, for instance when studying the structure of photosystem II and related model complexes with EXAFS.^497–499^

Jacob and co-workers proposed a general framework for quantifying uncertainties stemming from structural distortions of the input structure in theoretical spectroscopy. As quantity of interest, they chose the (discretized) spectral intensity as a function of the energy (usually obtained by applying an empirical broadening to the calculated individual transitions) instead of individual transitions themselves.^500^ This choice is motivated by the observation that for many types of spectroscopy, there are various close-lying transitions which cannot be resolved in experiment.

Because of the large number of degrees of freedom for molecular structures (3*N* – 6 for nonlinear molecules, where *N* is the number of atoms), assessing the structural sensitivity in a local sensitivity analysis is a high-dimensional problem. Bergmann et al.^501^ showed that by means of a principal component analysis in combination with a high-dimensional model representation (Sobol’ expansion), it becomes possible to construct a low-dimensional surrogate model, that can then be used to efficiently analyze the propagation of uncertainties due to structural distortions, and to assign corresponding error bars to the calculated spectra. This general framework has been applied in the calculation of X-ray emission, UV/vis, and infrared spectra. For related work on surrogate models for describing the structural sensitivity of X-ray spectra, see refs 502 and 503.

##### Vibrational Spectroscopy

In computational vibrational spectroscopy, it is common practice to apply scaling factors to calculated vibrational frequencies, that correct for errors in the underlying quantum-chemical method as well as additional approximations (such as the commonly applied harmonic approximation).^504–507^ These scaling factors are determined using a linear fit of calculated frequencies to experimental reference data.

Irikura et al. applied uncertainty quantification to scaling factors for harmonic vibrational frequencies^508^ and to vibrational zero-point energies^509^ by employing the standard deviation of this linear fit. Johnson et al. extended this work to scaling factors for anharmonic frequencies calculated with second-order vibrational perturbation theory.^510^ A case study for the X3LYP functional is presented in ref 511. A discussion of methodological aspects can be found in refs 512–514 .

Parks et al.^272^ applied the BEEF-vdW xc functional (see section 4.1.2) to calculate harmonic vibrational spectra, and propagated the uncertainties provided within the BEEF framework to the harmonic frequencies. They present an in-depth analysis of the resulting uncertainties, and find that certain types of vibrations (e.g., bending and torsional modes) are prone to higher uncertainties. They further point out that in many cases, in particular for intermolecular complexes, the ensembles obtained for the vibrational frequencies are non-Gaussian.

The calculation of anharmonic vibrational spectra generally requires solving the nuclear Schrödinger equation on the full potential energy surface (for reviews, see refs 515–518 ). Such calculations are extremely challenging, and the accuracy of the final vibrational transition energies and possibly intensities is intricately determined by multiple sources of errors, such as the accuracy of the underlying potential energy surface (including its representation in suitable coordinates^519–522^ ) and the solution of the nuclear Schrödinger equation using vibrational correlation methods.^523–525^ Addressing the latter, Larsson^526^ established error bound for the individual vibrational energy levels obtained with a given potential-energy surface of acetonitrile. To this end, they performed an extrapolation with respect to the bond dimension *D* in their tree tensor network calculations. These uncertainty estimates do, however, not account for uncertainties in the potential energy surface. Some steps toward quantifying the uncertainties related to both the potential energy surface and the solution of the nuclear Schrödinger equation have been made by König and Christiansen.^527^

##### UV/Vis Spectroscopy and Photodynamics

There are only a few studies that are related to the quantification of uncertainties in quantum-chemical calculations of electronic excitations. These consider the uncertainties due to the quantum-chemical approximations with respect to experimental data or to high-level computational reference data.

In an early study, Edwards et al.^528^ considered the prediction of vertical ionization potentials (IPs) of small water clusters with a double-hybrid xc functional. For the water dimer, trimer, tetramer, and pentamer, they determined intervals for the fraction of Hartree–Fock exchange and of MP2 correlation that lead to predictions that are consistent with high-level reference data within the Bound-to-Bound Data Collaboration framework.^315–317^ Subsequently, they use these intervals for those two parameters of the xc functional to establish an error interval for the vertical IP of different isomers of the water hexamer.

The accuracy of TD-DFT predictions of the energies of electronically excited state as well as of the corresponding transition moments widely varies with the employed xc functionals, and the accuracy of different xc functionals is highly dependent on both the considered molecules and on the nature of the relevant excited states. Avagliano et al.^529^ employed a huge dataset containing the five lowest singlet excited states for over 20,000 molecules to assign a score to each of 38 xc functional. This score combines the uncertainties in the one-electron transition density matrix, the excited states energies, and the transition dipole moments. They then trained a graph attention neural network to predict these scores from the molecular structure, and to recommend the xc functional with the best expected performance for this specific molecule.

In simulations of the nonadiabatic dynamics following photoexcitations, the outcome of the simulations can sensitively depend on the underlying ground and excited state potential energy surfaces. Jíra et al.^530^ investigated the photochemistry of *cis*-stilbene using trajectory surface hopping methods and found a large dependence of the quantum yields for two different products on the underlying electronic structure method. They suggest the use of a biasing potential, which allows one to efficiently quantify this sensitivity.

##### X-ray Spectroscopy

In many cases, the calculation of spectra involves additional approximations on top of the quantum-chemical methods that are employed. One such an example is computational X-ray spectroscopy, where one commonly applies the core–valence separation approximation, in which excitations from core orbitals are separated from excitations from valence orbitals. To assess the error arising from the approximation, Herbst and Franson^531^ developed a postprocessing step for ADC(2) calculations employing the core–valence separation. They apply Rayleigh quotient iterations to refine the resulting eigenvectors, and to assess the corresponding error in the eigenvalues.

X-ray absorption spectra mainly probe the local environment of the absorbing atom. Therefore, machine learning models that are trained to predict them from descriptors of this local chemical environment have been developed. Ghose et al. put forward uncertainty quantification for such models by training neural network ensembles^532^ (also refer to section 4.1.5), considering the N, O, and C K-edge spectra of the small organic molecules in the QM9 dataset. Conversely, neural networks can be trained to classify which structural elements are present in a molecule form its X-ray absorption spectrum. Again, a neural network ensemble can be used to provide uncertainties for such a classifier.^533^ For the Fe K-edge X-ray absorption spectra, Verma et al. trained deep neural networks, and estimated the uncertainty of their predictions using bootstrap resampling^534^ (see section 3.4.2).

##### Mössbauer Spectroscopy

Most studies on theoretical Mössbauer spectroscopy focus on the isomer shift (via the contact density) and/or the quadrupole splitting (via the electric-field gradient), both of which are spectral features caused by electric hyperfine interactions.^535^

Proppe and Reiher^536^ were the first to study the problem from a UQ perspective and applied a range of statistical tools, including bootstrapping (section 3.4.2) and Bayesian inference (section 3.2.2), to estimate confidence intervals for isomer shifts derived from DFT-computed contact densities of iron complexes. These confidence intervals take into account both residuals (model error) and fluctuations in regression coefficients (parameter uncertainty). They found that the average predicted confidence interval is significantly larger than the average experimental uncertainty. More complex (i.e., nonlinear) fitting functions could not resolve this issue. An outlier analysis based on the jackknife-after-bootstrapping method^537,538^ revealed that five of the 44 studied complexes are responsible for the disagreement. After removal of these outliers, the average predicted confidence interval became representative of the average experimental uncertainty.

As pointed out later by Krewald and co-workers,^539^ a valid UQ model is not enough to obtain reliable predictions of Mössbauer parameters. If the electronic structure is qualitatively wrong, e.g., due to non-negligible multireference character, predictions of Mössbauer parameters will be compromised by biased contact densities and electric-field gradients. It is therefore crucial—not only for this type of application—to validate the input fed into a UQ model or any other kind of prediction model. Referring to the example above, one would not know that the five outliers were actually outliers without further analysis if they had not been part of the regression procedure.

The UQ method developed by Proppe and Reiher^536^ for computational Mössbauer spectroscopy was refined in the work by Krewald and co-workers^539^ and has been applied, for instance, to study how myoglobin-catalyzed azide reduction proceeds via an anionic metal amide intermediate.^540^

#### 4.3.4 Quantum Cluster Equilibrium Method

In the framework of the quantum cluster equilibrium (QCE) theory, the liquid phase is described by an ensemble of interacting gas phase molecular clusters. Thermodynamic properties of the bulk phase are accessible by combining static quantum-chemical calculations of an ensemble of clusters, differing e.g., in the number of molecules, conformation, composition, etc., with simple statistical mechanics and subsequent cluster weighting or population analysis.^541–543^ By assuming that the clusters are in equilibrium with each other and relying on mass conservation, the total partition function can be expressed, providing access to thermodynamic functions, such as the energy, entropy, and enthalpy. The QCE theory has been applied to calculate thermodynamic quantities of many condensed phase systems,^544–547^ and cluster weighting was further employed to obtain spectroscopic^548–551^ data or conductivities^552,553^ in the condensed phase. In particular, vibrational circular dichroism spectra, which strongly depend on the conformational sampling could be accessed.^554^ Recently, a multicomponent QCE theory has been developed, allowing to describe any type of liquid mixtures.^555^

QCE was also used to study uncertainty quantification by Blasius et al.^556^ In that work, the dependence of the vaporization enthalpies and entropies of several organic liquids on the uncertainties in the experimental QCE input data was investigated. The vaporization enthalpies and entropies showed a smooth dependence on changes in the reference density and boiling point, and while the density showed little influence on the vaporization thermodynamics, variations in the input boiling point had a larger effect on the vaporization enthalpy, but only little effect on the vaporization entropy. Next, a quantification of uncertainty in thermodynamic functions that originates from inaccuracies in the experimental reference data via the Gauss–Hermite estimator was carried out, finding an uncertainty of 30.95 kcal mol^–1^ for (*R*)-butan-2-ol.

#### 4.3.5 Machine Learning of Static Properties

In recent years, a variety of approaches have been developed to quantify and validate uncertainty in machine learning models for molecular property prediction. Ensemble-based methods (also refer to MLIPs in section 4.1.5) and calibration-oriented methods form one major direction in this context. Busk et al.^557^ introduced a framework for calibrated uncertainty estimation in message passing neural networks (MPNNs) trained on QM9 ^558^ and PC9.^559^ They demonstrated that recalibration via isotonic regression improves the reliability of aleatoric and epistemic uncertainty estimates, with validation on out-of-distribution data using the expected normalized calibration error (ENCE). Similarly, Gruich et al.^560^ compared *k*-fold ensembling, Monte Carlo dropout, and evidential regression for crystal graph convolutional neural networks (CGCNNs) trained on the Open Catalyst 2020 dataset, showing that evidential regression combined with scalar recalibration yields particularly trustworthy uncertainty estimates for adsorption energies in catalysis. Both studies highlight the importance of calibration for ensuring meaningful uncertainty estimates in chemical machine learning.

A number of works provide systematic analyses and benchmarking studies across different UQ techniques. Heid et al.^561^ evaluated ensembles, mean–variance estimation, and conformal prediction using various neural network architectures, including d-MPNNs and SchNet,^562^ focusing on the decomposition of epistemic uncertainty into bias and variance contributions. Hirschfeld et al.^563^ extended this perspective by benchmarking ensemble-, mean-variance-, distance-, and union-based UQ approaches on multiple datasets, finding that dataset-specific factors strongly influence UQ performance. These studies underscore that no single UQ method is universally superior and that calibration quality and data characteristics are crucial determinants of reliability.

Several works explored Bayesian or probabilistic formulations of uncertainty estimation (see section 3.2.2). Janet and Kulik^564^ applied Monte Carlo dropout and Gaussian-process-based variance estimation to neural networks predicting DFT-derived properties of transition metal complexes, demonstrating that such uncertainty estimates help identify unreliable predictions. Ryu et al.^565^ proposed a Bayesian graph convolutional network (GCN) to separate epistemic and aleatoric contributions, showing improvements in model reliability for datasets covering bioactivity and photovoltaic properties. These methods illustrate how Bayesian inference can naturally incorporate data and model uncertainty in chemical learning tasks.

Latent-space and distance-based methods have been developed as computationally efficient alternatives. Janet et al.^566^ introduced a latent space distance metric for neural networks that correlates well with predictive error while outperforming ensemble and dropout methods in calibration. Zhang et al.^567^ further applied similarity-based pairing in Siamese neural networks to derive variance-based uncertainty measures, confirming that confidence scores derived from pairwise similarity correspond to actual prediction reliability. Together, these works demonstrate that representations learned by neural networks can provide valuable internal indicators of prediction uncertainty.

In addition to these approaches, uncertainty quantification has been integrated into Gaussian process models (see also section 3.5.2). Musil, Ceriotti, and co-workers^334^ proposed a subsampling-based estimator for Gaussian process regression (GPR) that reproduces reliable UQ at lower computational cost compared to standard GPR variance estimation. Wollschläger et al.^568^ extended this idea by introducing the localized neural kernel (LNK), a Gaussian-process-inspired extension to graph neural networks, achieving improved calibration and out-of-distribution detection in molecular force-field modeling. These methods illustrate how kernel-based approaches can provide interpretable and computationally tractable uncertainty estimates.

Several studies focused explicitly on evaluating or validating UQ metrics (section 3.6). Rasmussen et al.^569^ systematically compared different UQ validation metrics for chemical ML and concluded that error-based calibration analysis offers a more reliable assessment of UQ performance than traditional correlation-based measures. Scalia et al.^570^ performed a large-scale comparison of deep ensembles, Monte Carlo dropout, and bootstrapping across chemical and biochemical datasets, finding that ensemble-based approaches generally achieve superior calibration. Both works emphasize that the choice of evaluation metric is crucial for assessing uncertainty quality.

Finally, a number of specialized approaches extend uncertainty quantification beyond global property prediction. Tynes et al.^571^ introduced pairwise difference regression (PADRE), which reformulates regression tasks into pairwise comparisons, providing intrinsic uncertainty estimates that improve candidate selection. Yang and Li^572^ developed an atom-based uncertainty method combining deep ensembles with posthoc calibration, enabling atomic-level attribution of uncertainty and improving interpretability.

A promising application of UQ in ML of static properties are multifidelity methods, in which high-quality training data is combined with cheaper and less accurate training data to achieve the accuracy of the costlier level.^573,574^ Such methods have successfully been employed for ML models of atomization energies,^573^ band gaps,^575,576^ potential energy surfaces,^574^ and of spectroscopic properties.^577^

Overall, these studies collectively reveal a landscape of complementary approaches to uncertainty quantification in molecular machine learning, spanning ensemble-based calibration, Bayesian inference, latent space metrics, kernel-based methods, and specialized architectures designed for interpretability or data efficiency.

## 5 Conclusion

In this review, we have presented an overview of applications of UQ for *in silico* chemistry. Mathematical tools developed in applied mathematics for quantifying uncertainties in computational sciences have been increasingly adopted in chemistry in recent years. The methods and applications discussed in this review have the potential to transform *in silico* chemistry by providing reliable uncertainties for a wide range of quantities of interest. The gained insights will increase the reliability of predictions from *in silico* methods in comparison to experiments.

A particularly important goal of UQ in quantum chemistry is to establish uncertainty estimates in (relative) energies of quantum-chemical calculations for specific molecules. Many different sources of uncertainty in such calculations have been tackled using a wide range of methods. Established tools, such as basis set extrapolation schemes, indicators of multireference character, or automated schemes for the selection of the active space in multireference calculations, can be reinterpreted as uncertainty estimates. However, they will require further validation to arrive at routinely applicable tools. Similarly, developments such as xc functionals, including uncertain parameters, still need to find their way into routine applications of quantum-chemical program packages.

We expect particularly large benefits from further developments of UQ in quantum chemistry in the field of multilevel and multifidelity methods, where uncertainty estimates can play a decisive role in guiding the partitioning of complex chemical systems into subsystems. Moreover, selecting suitable methods for each subsystem in such a way that a quantity of interest (e.g., an enzymatic reaction energy or spectroscopic properties of a chromophore embedded in a complex environment) is as accurate as necessary for a specific chemical problem at hand.

With regard to MD simulations, two main targets have gained notable attention for developing and applying UQ, namely simulation parameters and trajectory analysis. Various mathematical methods, many of them in the context of local sensitivity analysis, have been employed to investigate the propagation of uncertainties in MD input parameters, covering both force field parameters as well as other parameters such as the system size, simulation time, or temperature. Efforts have been made to transform traditional approaches of finding optimal sets of parameters, which often lack reproducibility and a systematic estimation of uncertainties, into UQ-guided, mathematically sound workflows.

Additionally, UQ has been incorporated into postprocessing routines for MD simulations, particularly in the context of free energy calculations, and correlation functions. In that context, replica simulations, in conjunction with methods that enhance or improve sampling of the phase space, thermodynamic functions and other QoIs, have proven instrumental for yielding accurate and precise simulation predictions with interpretable uncertainties. We anticipate that alongside the growing interest of UQ for MD simulations, more advanced UQ tools, such as global sensitivity analysis and multifidelity techniques will be developed for and applied to complex QoIs.

In ML, UQ has led to a diverse set of methodological approaches, and the field is still evolving and far from being uniformly established. In chemical applications of ML, most importantly in MLIPs, uncertainty estimates are particularly useful for indicating whether the predictions of a ML model are within the domain of its training data. This is in turn heavily exploited in active learning strategies that can reduce the number of data points required for training. As the training data is usually derived from expensive quantum-chemical calculations, the economical use of training data that is achieved by leveraging UQ is particularly relevant for *in silico* chemistry.

A particularly attractive application of ML in UQ for *in silico* chemistry, with vast potential for new method development, is the use as surrogate models in multifidelity approaches. For example, by training Δ-ML models of the errors of computational predictions (e.g., with respect to experimental data or to high-level computational reference data), it becomes possible to provide error estimates, and to select computational models (e.g., xc functionals) that are most suitable for a specific problem at hand (e.g., the calculation of excitation energies).

ML encompasses a broad range of model classes with different parametrizations, from comparatively low-parametric models to highly parametrized deep neural networks. In many contemporary applications, particularly those based on deep neural networks, the extremely high dimensionality of the parameter space renders classical parameter-based uncertainty analyses impractical, motivating alternative approaches that focus on predictive uncertainty and robustness. A particularly critical and still unresolved challenge is the reliable quantification of uncertainty for inputs that differ substantially from the training data, where predictions rely on extrapolation rather than interpolation.

For many ML models, especially those used in chemistry and materials science, UQ is more naturally formulated at the level of predictions rather than model parameters, which often lack direct physical meaning. Even if an effective reduction of the parameter space can be achieved, this does not automatically translate into actionable insight for improving the trustworthiness of predictions in practical applications. The reliability of uncertainty estimates in ML models is further conditioned on implicit structural assumptions about the data and models, such as smoothness or differentiability, which are often difficult to validate in realistic settings.

Stochastic effects arising from random initialization can lead to differently trained models even when architectures and training data are identical. Ensemble-based approaches are often used to capture this variability at the level of predictions.

Summarizing the applications of UQ in *in silico* chemistry reviewed here, it is apparent that most previous work focused on UQ of a specific source of uncertainty in a specific chemical application. Given the wide range of both, many previous works have come up with *ad hoc* approaches in each case. Therefore, it becomes hard to spot the universality across these approaches to UQ in *in silico* chemistry. While this is to some extent a consequence of the wide range of computational methods that are relevant in chemistry, we expect that the next years will see a consolidation of different approaches to UQ in *in silico* chemistry. The development of user-friendly, open-source UQ software that provide broadly applicable UQ methods with applicability to *in silico* chemistry could catalyze such a consolidation and incentivize the community to adopt such of-the-shelf approaches to UQ.

So far, most of the work reviewed here has tackled the quantification of a single, specific source of uncertainty. The classification of sources of uncertainty in chemical applications and the identification of the most relevant sources of uncertainty remains an important open topic, given the diversity of *in silico* chemistry. Future work across all areas of *in silico* chemistry will further need to combine UQ of different sources of uncertainties. On that account, the development of UQ methods that are able to account for systematic error compensation between different sources of errors—on which computational chemistry heavily relies—seems to be a particularly promising avenue.

While the past years have seen tremendous progress in the development of UQ methods for *in silico* chemistry, best practices for UQ in different types of applications will need to be established and adopted by researchers. First steps toward establishing UQ as an integral requirement of computational workflows have been started in the molecular dynamics community.^65^

We conclude by emphasizing that uncertainty quantification is far more than “just statistics”: it provides rigorous frameworks that have advanced *in silico* chemistry and will remain central to its future development.

## Footnotes

a Some mathematical subtleties have to be taken into account,^51,104^ but these are of no importance to most readers.

b Taking care of a few mathematical subtleties,^51^ most of the things can be generalized beyond the case $\Omega \subset R^{d}$ .
